# The slow-scale linear noise approximation: an accurate, reduced stochastic description of biochemical networks under timescale separation conditions

**DOI:** 10.1186/1752-0509-6-39

**Published:** 2012-05-14

**Authors:** Philipp Thomas, Arthur V Straube, Ramon Grima

**Affiliations:** 1 Department of Physics, Humboldt University of Berlin, Berlin, Germany; 2 School of Biological Sciences, University of Edinburgh, Edinburgh, UK; 3 SynthSys Edinburgh, University of Edinburgh, Edinburgh, UK

## Abstract

**Background:**

It is well known that the deterministic dynamics of biochemical reaction networks can be more easily studied if timescale separation conditions are invoked (the quasi-steady-state assumption). In this case the deterministic dynamics of a large network of elementary reactions are well described by the dynamics of a smaller network of effective reactions. Each of the latter represents a group of elementary reactions in the large network and has associated with it an effective macroscopic rate law. A popular method to achieve model reduction in the presence of intrinsic noise consists of using the effective macroscopic rate laws to heuristically deduce effective probabilities for the effective reactions which then enables simulation via the stochastic simulation algorithm (SSA). The validity of this heuristic SSA method is *a priori* doubtful because the reaction probabilities for the SSA have only been rigorously derived from microscopic physics arguments for elementary reactions.

**Results:**

We here obtain, by rigorous means and in closed-form, a reduced linear Langevin equation description of the stochastic dynamics of monostable biochemical networks in conditions characterized by small intrinsic noise and timescale separation. The slow-scale linear noise approximation (ssLNA), as the new method is called, is used to calculate the intrinsic noise statistics of enzyme and gene networks. The results agree very well with SSA simulations of the non-reduced network of elementary reactions. In contrast the conventional heuristic SSA is shown to overestimate the size of noise for Michaelis-Menten kinetics, considerably under-estimate the size of noise for Hill-type kinetics and in some cases even miss the prediction of noise-induced oscillations.

**Conclusions:**

A new general method, the ssLNA, is derived and shown to correctly describe the statistics of intrinsic noise about the macroscopic concentrations under timescale separation conditions. The ssLNA provides a simple and accurate means of performing stochastic model reduction and hence it is expected to be of widespread utility in studying the dynamics of large noisy reaction networks, as is common in computational and systems biology.

## Background

Biochemical pathways or networks are typically very large. A well-characterized example is the protein-protein interaction network of the yeast *Saccharomyces cerevisiae* with approximately a thousand putative interactions involving an approximate equal number of proteins [1]. It is also a fact that a significant number of species are found in low copy numbers in both prokaryotic and eukaryotic cells [2,3]. Recent mass spectrometry-based studies have, for example, shown that 75% of the proteins in the cytosol of the bacterium *Escherichia coli* appear in copy numbers below 250 and the median copy number of all identified proteins is approximately 500 [3]. This means that simulation methods intended to realistically capture the inner workings of a cell have to (i) be stochastic to take into account the significant intrinsic noise associated with low copy number conditions; (ii) be able to simulate fairly large networks in a reasonable amount of time. The stochastic simulation algorithm (SSA) [4] has been and still is the algorithm of choice for a large number of studies exploring the role of noise in biology. The advantage of the algorithm is that it is exact, i.e., it exactly samples the trajectories of the stochastic process described by the chemical master equation (CME), the accepted mesoscopic description of chemical kinetics. Its disadvantage is that it simulates every reaction event and hence is not particularly suited for the study of large networks [5]. This problem is an outstanding challenge in the fields of computational and systems biology.

A common way of circumventing the problem is to simulate a network of species which is much smaller than the size of the full network but which nevertheless captures the essential dynamics. For example, the three elementary (unimolecular or bimolecular) reactions which describe the enzyme-assisted catalysis of substrate *S* into product *P* via the Michaelis-Menten reaction, $S\;\;+\;\;E\;\overset{k_{0}}{\underset{k_{1}}{⇌}}\;C\overset{k_{2}}{\to }E\;+\;P$, can be replaced by a single effective reaction $S\overset{k^{\prime }}{\to }P$. Note that here *E* and *C* denote the free enzyme species and the enzyme-substrate complex species, respectively. The latter first-order reaction is non-elementary, i.e., it can be broken down into a set of fundamental elementary reactions. The implicit assumption in this lumping or coarse-graining method is that the transients in the average concentrations of some species decay over much longer timescales than those of the rest of the species. Hence, one can argue that the relevant network to be simulated is that involving the slowly varying species only. In the Michaelis-Menten example, the fast species were the enzyme and the complex and the slow species are the substrate and product. The dynamics of this reduced network are of course only a faithful approximation of those of the full network, the Michaelis-Menten reaction, whenever the rate constants guarantee reasonable timescale separation.

On the macroscopic level, where molecule numbers are so large that intrinsic noise can be ignored, there is a well-known practical recipe for obtaining this reduced or coarse-grained network from the full network of elementary reactions. One writes down the rate equations (REs) for each species, decides which species are fast and slow, sets the time derivative of the concentration of the fast species to zero, solves for the steady-state concentrations of the fast species and finally substitutes these concentrations into the equations for the slow species. This procedure is the deterministic quasi-steady-state assumption (QSSA). The result is a set of new REs for the slow species only; corresponding to these reduced equations is the coarse-grained network, i.e., the network of reactions between slow species whose macroscopic rate laws are dictated by the new REs. Generally, all coarse-grained networks will have at least one reaction which is non-elementary; however those reactions involving the interaction of only slow species in the full network will naturally also remain elementary in the coarse-grained network. The deterministic QSSA presents a rigorous method of achieving a coarse-grained macroscopic description based on the deterministic REs [6]. Its major shortcoming is that it ignores the inherent stochasticity of the system.

On the mesoscopic level, or, in other words, whenever the size of intrinsic noise becomes comparable with the average molecule numbers, the description of chemical kinetics is given by the CME. One would hope that under conditions of timescale separation, just as one can write effective REs for a coarse-grained network starting from the REs of the full network, in a similar manner one can obtain an effective (or reduced) CME for the coarse-grained network starting from the CME of the full network. The effective REs have information about the macroscopic concentrations of the slow species only, while the effective CME has information about the fluctuations of the slow species only. This line of reasoning has led to a stochastic formulation of the QSSA which is in widespread use. In what follows we concisely review the CME formulation of stochastic kinetics and point out compelling reasons which cast doubt on the validity of the popular stochastic QSSA.

Suppose the network (full or coarse-grained) under consideration consists of a number *N* of distinct chemical species interacting via *R* elementary or non-elementary chemical reactions of the type

(1) $$s_{1j}X_{1}+\ldots +s_{\mathrm{Nj}}X_{N}\overset{k_{j}}{\to }\;r_{1j}X_{1}+\ldots +r_{\mathrm{Nj}}X_{N}.$$

**Figure 1:**
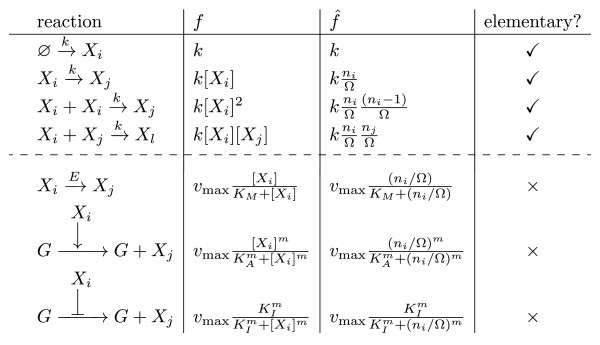
**Microscopic and macroscopic rate functions.** The macroscopic rate function *f * and the microscopic rate function $\left(\hat{f}\right)$ for various common types of chemical reaction steps. The former define the REs while the latter define the CME. The first four reactions are elementary, i.e., they are unimolecular or bimolecular reactions. The last three reactions are non-elementary, i.e., they can be decomposed into a number of simpler elementary reactions. These reactions represent (from top to bottom) the catalysis of a substrate by enzyme, up-regulation of a gene (G) by an activator and down-regulation of a gene by a repressor

Here, *j* is an index running from 1 to *R*, *X*_*i*_denotes chemical species *i*, *s*_*ij*_and *r*_*ij*_ are the stoichiometric coefficients and *k*_*j*_is the macroscopic rate coefficient of the reaction. If reaction scheme (1) describes the full network with *N*_*s*_ number of slow species and *N*_*f*_=*N*−*N*_*s*_ number of fast species, then we adopt the convention that *X*_1_to $\left(X_{N_{s}}\right)$ denote the slow species, while $\left(X_{N_{s}+1}\right)$ to *X*_*N*_ label the fast species. Let *n*_*i*_denote the absolute number of molecules of the *i*th species; then, at any point in time, the system is described by the state vector $\vec{n}={\left(n_{1},\ldots ,n_{N}\right)}^{T}$. When the *j*th reaction occurs, the system jumps from state $\left(\vec{n}\right)$ to a new state $\vec{n}+{\vec{\mu }}_{j}$, where ${\vec{\mu }}_{j}=\left(r_{1j}-s_{1j},\mathrm{...},r_{\mathrm{Nj}}-s_{\mathrm{Nj}}\right)$. Furthermore, one defines a propensity function *a*_*j*_ for the *j*th reaction such that $a_{j}\left(\vec{n}\right)\mathrm{dt}$ is the probability that the *j*th reaction occurs in the next infinitesimal time interval $\left[t,t+\mathrm{dt}\right)$. Using these definitions and the laws of probability, one can then deduce that the general form of the CME is [5]

(2) $$\;\frac{\mathrm{\partial P}\left(\vec{n},t\right)}{\mathrm{\partial t}}=\overset{R}{\underset{j=1}{\sum }}\left[a_{j}\left(\vec{n}-{\vec{\mu }}_{j}\right)P\left(\vec{n}-{\vec{\mu }}_{j},t\right)-a_{j}\left(\vec{n}\right)P\left(\vec{n},t\right)\right],$$

where $\left(P(\vec{n},t)\right)$ is the probability that the system is in a particular mesoscopic state $\vec{n}$. The recipe becomes complete once we specify the form of the propensity functions for each chemical reaction. Figure 1 lists the microscopic rate function, ${\hat{f}}_{j}=a_{j}/\Omega$, i.e., the propensity functions divided by the volume Ω, for 4 elementary reactions and 3 common non-elementary reactions. The macroscopic rate function *f*_*j*_, i.e., the rate of reaction according to the deterministic REs, is also shown alongside the microscopic rate functions. Note that $\left[X_{i}\right]$ in the macroscopic rate functions denotes the macroscopic concentration of species *i*.

If we are modeling the full network, then the constituent reactions have to be all elementary. For such reactions, the propensity and microscopic rate functions have been derived from molecular physics [7] and hence the CME for the full network is fundamentally correct. Now say that we are modeling a coarse-grained network in which case some reactions are non-elementary. Microscopic considerations do not tell us anything about the form of the propensity functions for such reactions. Rather the propensities and the microscopic rate functions are a heuristic extrapolation of the macroscopic reaction rates at the heart of the effective REs for the non-elementary reactions: $\left(\hat{f}\right)$ is obtained by performing the substitution $\left[X_{i}\right]\to n_{i}/\Omega$ on *f *.

*Hence it follows that the CME for the coarse-grained network is not rigorously derived from that of the full network under conditions of timescale separation but rather is heuristic and hence its validity is a priori doubtful*. Janssen was the first to investigate this question by means of an analytical approach applied to a simple chemical example, the dissociation of *N*_2_*O*_5_; he showed that “the master equation for a complex chemical reaction cannot always be reduced to a simpler master equation, even if there are fast and slow individual reaction steps” [8]. This suggests that even if the molecules numbers are quite large, the conditions for timescale separation required for the validity of the deterministic QSSA are not generally enough to guarantee the validity of the heuristic CME, a hypothesis which has been recently verified in the context of the Michaelis-Menten reaction with substrate input [9]. In other words, *the heuristic CME is not the legitimate stochastic equivalent of the deterministic QSSA, in the sense that it does not correctly describe the statistics of the intrinsic noise about the macroscopic concentrations as given by the reduced REs of the coarse-grained network*.

Notwithstanding the fundamental objections of Janssen, and frequently in the name of pragmatism, many studies [10-13] have employed the heuristic CME to obtain a coarse-grained stochastic description of various complex networks. A number of studies [14-17] have reported good agreement between the results of stochastic simulations of the full and coarse-grained networks for enzyme reactions and circadian oscillators which has enhanced faith in the heuristic approach of stochastic modeling of networks with non-elementary reactions and given it the status of a mainstream methodology.

In this article we seek to derive a rigorous alternative to the heuristic approach. Given the CME of the full network of elementary reactions, we derive a reduced linear Fokker-Planck equation (FPE) which describes the noise statistics of the same network when the molecule numbers are not too small and under the same conditions of timescale separation imposed by the deterministic QSSA. This new FPE is the legitimate mesoscopic description of intrinsic noise about the macroscopic concentrations of the coarse-grained network as obtained by the deterministic QSSA. The noise statistics from this approach are compared with stochastic simulations of the full network and with simulations of the coarse-grained network using the conventional heuristic approach. In all cases our approach agrees very well with the full network results. In contrast, we show how the size of intrinsic noise as predicted by the conventional approach can be different by more than an order of magnitude than the actual value and how in some instances this approach even misses the existence of noise-induced oscillations. We also show using our method how one can obtain the regions of parameter space where the conventional approach qualitatively fails and where it fares well.

The article is organized as follows. In the Results section, we discuss in general terms the procedure of obtaining a rigorous mesoscopic description under conditions of timescale separation akin to those of the deterministic QSSA. We then apply this novel method to two different examples: an enzyme mechanism capable of displaying both Michaelis-Menten and Hill-type kinetics and a gene network with a single negative feedback loop. The results from our method are contrasted and compared with stochastic simulations of the full network and with those of the coarse-grained network using the conventional heuristic method. We finish by a discussion. Detailed derivations concerning results and applications can be found in the Methods section.

## Results

The optimal method to determine the validity of the heuristic CME would be to obtain its analytical solution and compare it with that of the CME for the full network and for rate constants chosen such that the deterministic QSSA is valid. Note that the latter constraint on rate constants is necessary because the propensities of the heuristic CME are based on the macroscopic rate laws as given by the reduced REs and hence the heuristic CME can only give meaningful results if the deterministic QSSA is valid. Unfortunately, CMEs are generally analytically intractable, with exact solutions only known for a handful of simple elementary reactions [18-20]. To circumvent this problem we take recourse to a systematic approximation method, the system-size expansion of van Kampen [21]. The starting point of this method is to write the absolute number of molecules of species *i* in the CME, equation (2), as

(3) $$\frac{n_{i}}{\Omega }=\left[X_{i}\right]+\Omega^{-1/2}\varepsilon_{i},$$

where $\left[X_{i}\right]$ is the macroscopic concentration of species *i* and *ε*_*i*_is proportional to the noise about this concentration. This substitution leads to an infinite expansion of the master equation. The first term, that proportional to Ω ^1/2^, leads to the deterministic equations for the mean concentrations as predicted by the CME in the macroscopic limit of large volumes (or equivalently large molecule numbers). The rest of the terms give a time-evolution equation for the probability density function of the fluctuations, $\Pi (\vec{\varepsilon },t)$. This partial differential equation is an infinite series in powers of the inverse square root of the volume (see [22] for the general form of this equation). Truncating this series to include only the first term, i.e., that which is proportional to Ω ^0^, leads to a second-order partial differential equation, also called the linear Fokker-Planck equation or the linear noise approximation (LNA) [21,23,24]. The solution of the latter equation is a multivariate Gaussian probability distribution and hence expressions for the statistics of intrinsic noise about the macroscopic concentrations, e.g., the variance of fluctuations, can be obtained straightforwardly from this formalism, a distinctive advantage over the CME. The restrictions which must be kept in mind are that this method only provides a reliable approximation to the CME if the molecule numbers are sufficiently large (small intrinsic noise) and the chemical network is monostable (see also the Discussion and conclusion section).

Hence we can now formulate two questions to precisely determine the validity of the heuristic CME in timescale separation conditions: (i) in the macroscopic limit, are the mean concentrations of the heuristic CME exactly given by the reduced REs obtained from the deterministic QSSA? (ii) are the noise statistics about these mean concentrations, as given by the LNA applied on the heuristic CME, equal to the noise statistics obtained from applying the LNA on the CME of the full network? If the heuristic CME is correct then the answer to both these questions should be yes.

The first question can be answered straightforwardly. The deterministic equations for the mean concentrations of the heuristic CME, in the macroscopic limit of infinite volumes, necessarily only depend on the macroscopic limit of the heuristic microscopic rate functions in the heuristic CME. More specifically, consideration of the first term of the system-size expansion leads to a deterministic set of equations of the form $d\vec{\left[X\right]}/\mathrm{dt}=S\;\;\vec{\hat{f}}{|}_{\Omega \to \infty }$, where S is the stoichiometric matrix with elements *S*_*ij*_=*r*_*ij*_−*s*_*ij*_[23]. As discussed in the Introduction, the vector of heuristic microscopic rate functions for the heuristic CME, $\left(\vec{\hat{f}}\right)$, is constructed from the macroscopic rate function vector, $\vec{f}$, of the reduced REs by performing the substitution $\left[X_{i}\right]\to n_{i}/\Omega$ on $\vec{f}$. Given the ansatz, equation (3), we can see that the heuristic method guarantees, by construction, that $\vec{\hat{f}}{|}_{\Omega \to \infty }=\vec{f}$. This implies that the first term of the system-size expansion applied to the heuristic CME leads to a deterministic set of equations of the form $d\vec{\left[X\right]}/\mathrm{dt}=S\;\;\vec{f}$, which indeed are the reduced REs obtained from the deterministic QSSA. Hence, we can conclusively state that in the macroscopic limit, the heuristic CME does reproduce the correct mean concentrations for timescale separation conditions.

The second question, regarding agreement in noise statistics not simply in the means, has not been considered before and presents a considerably more difficult challenge. In what follows we briefly review the LNA applied to the heuristic CME of the coarse-grained network which we shall call the hLNA and we derive the LNA applied to the full network under conditions of timescale separation, a novel method which we refer to as the slow-scale LNA (ssLNA).

### The LNA applied to the heuristic CME

The application of the LNA to the heuristic CME has been the subject of a number of studies [23,25-30]. For a step-by-step guide to implementing the LNA we refer the reader to the supplementary material of Ref. [9]. Here we simply state the well known results. We shall use the underline notation to denote a matrix throughout the rest of the article.

Given the coarse-grained network, reaction scheme (1), one can construct the stoichiometric matrix S with elements *S*_*ij*_=*r*_*ij*_−*s*_*ij*_ and the macroscopic rate vector with entries $f_{j}=k_{j}\overset{N}{\underset{m=1}{\prod }}{\left[X_{m}\right]}^{s_{\mathrm{mj}}}$. Note that the latter, as discussed in the Introduction, encapsulates the macroscopic rate law for each individual reaction composing the network. Note also that the *k’s* for a coarse-grained network are generally functions of the macroscopic concentrations and not constants as for a full network (of elementary reactions). The reduced REs are then given by $d\vec{\left[X\right]}/\mathrm{dt}=S\;\;\vec{f}$ and consequently the Jacobian matrix J has elements $J_{\mathrm{ij}}=\partial_{j}{\left(\;S\;\;\vec{f}\right)}_{i}$, where *∂*_*j*_ denotes the partial derivative with respect to $\left[X_{j}\right]$, the concentration of species *j*.

It then follows by the LNA that the noise statistics given by the heuristic CME, i.e., equation (2) with heuristic propensities, in the limit of large molecule numbers, are approximately described by the following linear FPE

(4) $$\frac{\mathrm{\partial P}\left({\vec{\eta }}_{s},t\right)}{\mathrm{\partial t}}=\left(-\nabla_{s}^{T}J\;\;{\vec{\eta }}_{s}+\frac{1}{2}\nabla_{s}^{T}D{\;}_{h}\nabla_{s}\right)P\left({\vec{\eta }}_{s},t\right),$$

where ${\vec{\eta }}_{s}$ is the vector of concentration fluctuations about the macroscopic concentrations of the slow species, i.e., $\eta_{s,i}=(n_{i}/\Omega )-\left[X_{i}\right]$. Note that in the traditional approach due to van Kampen [21] one writes a linear FPE for the noise vector ${\vec{\varepsilon }}_{s}$ because of the form of the ansatz, equation (3) (the subscript *s* denotes slow species). Here we have instead chosen to write the FPE for ${\vec{\eta }}_{s}=\Omega^{-1/2}{\vec{\varepsilon }}_{s}$ since $\vec{\eta }$ is the true measure of fluctuations about the mean concentrations. The operator ∇_*s*_denotes the vector of derivatives with respect to components of the vector ${\vec{\eta }}_{s}$. The matrix D_*h*_ is the diffusion matrix which is given by the following formula

(5) $$D{\;}_{h}=\Omega^{-1}S\;\;F\;\;S^{T},$$

where F is a diagonal matrix whose non-zero diagonal entries are the elements of the macroscopic rate function vector $\vec{f}$, i.e., $F=\text{diag}\;(\vec{f})$.

The solution of the linear FPE, equation (4), is a multivariate Gaussian and hence noise statistics can be straightforwardly computed. The covariance matrix H of the concentration fluctuations about the steady-state concentrations, as described by the linear FPE, can be obtained by solving the Lyapunov equation [24,31]

(6) $$J\;\;H+H\;\;J^{T}+D{\;}_{h}=0,$$

where *H*_*ij*_=〈*η*_*s*,*i*_*η*_*s*,*j*_〉. The variance of the fluctuations of species *j* is hence given by the *j*th diagonal element of H. The power spectrum of the concentration fluctuations of the *j*th species is given by

(7) $$P_{j}(\omega )={\left[{\left(i\;I\;\omega +J\;\right)}^{-1}\underset{─}{D}{\;}_{h}{\left(-i\;I\;\omega +J^{T}\right)}^{-1}\right]}_{\mathrm{jj}},$$

where I is the identity matrix, *i* is the imaginary unit number and *ω* is the frequency.

Note that we have chosen to compute the variance and power spectrum as our noise statistics for the following reasons. The variance can be used to calculate the Fano factor (variance of fluctuations divided by the mean concentration) and the coefficient of variation (standard deviation of fluctuations divided by the mean concentration) [32]. The coefficient of variation provides a non-dimensional measure of the size of intrinsic noise, and is a particularly natural measure when the probability distribution solution of the CME is approximately Gaussian. The Fano factor multiplied by the volume provides another non-dimensional measure of the noise level: it gives the size of the fluctuations in the molecule numbers relative to that of a Poissonian distribution with the same mean number of molecules. Generally these measures provide different but complementary information and both have been reported in recent experiments [33,34]. Hence in this article we calculate both measures. We also calculate the power spectrum which gives the intensity of fluctuations at a given frequency; a peak in the spectrum indicates noise-induced oscillations [35], a phenomenon which is of importance in biochemical networks responsible for biological rhythms such as circadian clocks [36].

### The LNA applied to the full network under conditions of timescale separation

The LNA approach mentioned in the previous subsection works equally well if applied to the CME of the full network. This leads to a linear FPE of the form

(8) $$\frac{\mathrm{\partial P}\left(\vec{\eta },t\right)}{\mathrm{\partial t}}=\left(-\nabla^{T}J{\;}_{F}\;\vec{\eta }+\frac{1}{2}\nabla^{T}D{\;}_{F}\nabla \right)P\left(\vec{\eta },t\right),$$

where $\vec{\eta }=\left({\vec{\eta }}_{s},{\vec{\eta }}_{f}\right)$ and ${\vec{\eta }}_{s}$ and ${\vec{\eta }}_{f}$ are, respectively, the vectors of concentration fluctuations about the macroscopic concentrations of the slow and fast species. The operator ∇ denotes the vector of derivatives with respect to components of $\vec{\eta }$. The matrix J_*F*_ is the Jacobian of the REs of the full network, while the diffusion matrix D_*F*_ is given by equation (5) with S and F now equal to the stoichiometric matrix and the diagonal matrix of the macroscopic rate function vector for the full network, respectively.

Note that while equation (4) is based on the heuristic CME and therefore inherits all its problems, equation (8) has no such problems: it is derived from the CME of the full network of elementary reactions, which is fundamentally correct. Hence, ideally, we would obtain the multivariate Gaussian solution of the two linear FPEs, compare and then decide upon the validity of the heuristic CME. Unfortunately, this direct comparison is impossible because equation (4) gives a joint probability distribution function for the slow species only, whereas equation (8) leads to a joint probability density function for both slow and fast species.

In the Methods section we devise an adiabatic elimination method by which, starting from equation (8), we obtain a closed-form solution for a linear FPE that describes the time evolution of the joint probability density function of slow variables only. We call the reduced linear FPE obtained from this method, the slow-scale LNA. Our result can be stated as follows. Under conditions of timescale separation consistent with the deterministic QSSA, the noise statistics of the slow species according to the CME of the full network can be approximately described by the following linear FPE

(9) $$\frac{\mathrm{\partial P}\left({\vec{\eta }}_{s},t\right)}{\mathrm{\partial t}}=\left(-\nabla_{s}^{T}J\;\;{\vec{\eta }}_{s}+\frac{1}{2}\nabla_{s}^{T}D{\;}_{\mathrm{ss}}\nabla_{s}\right)P\left({\vec{\eta }}_{s},t\right).$$

Note that the matrix J is the Jacobian of the reduced REs of the coarse-grained network, indeed the same as in equation (4). However, the diffusion matrix D_*ss*_ is generally different than D_*h*_, which indeed proves the non-validity of the heuristic CME as a stochastic description of the coarse-grained network. We show that the new diffusion matrix is given by

(10) $$D{\;}_{\mathrm{ss}}=\Omega^{-1}\left(\;A-B\;\right){\left(\;A-B\;\right)}^{T},$$

where $A=S{\;}_{s}\sqrt{F}$ and $B=J{\;}_{\mathrm{sf}}J\;{\;}_{f}^{-1}S{\;}_{f}\sqrt{F}$. We have also used the following convenient definitions(11)

where S and J_*F*_ are the stoichiometric and Jacobian matrices of the full network and F is the diagonal matrix of the macroscopic rate function vector of the full network with the macroscopic concentrations of the fast species expressed in terms of the macroscopic concentrations of the slow ones. The matrices S and J_*F*_ are partitioned into sub-matrices of the following sizes: S_*s*_ is an *N*_*s*_×*R* matrix, S_*f *_ is an *N*_*f*_×*R* matrix, J_*s*_ is an *N*_*s*_×*N*_*s*_ matrix, J_*sf *_ is an *N*_*s*_×*N*_*f*_ matrix, J_*fs*_ is an *N*_*f*_×*N*_*s*_ matrix and J_*f *_ is an *N*_*f*_×*N*_*f*_ matrix. Note that *N*_*s*_and *N*_*f*_ are the number of slow and fast species respectively. Note also that the fact that the new diffusion matrix can be written in the form of equation (10) immediately implies that it is symmetric and positive semi-definite, two crucial properties of the diffusion matrices for all FPEs [21]. It also follows that the variance and power spectrum of the slow species according to the ssLNA can be calculated from equations (6) and (7) with D_h_ replaced by D_ss_.

**Figure 2:**
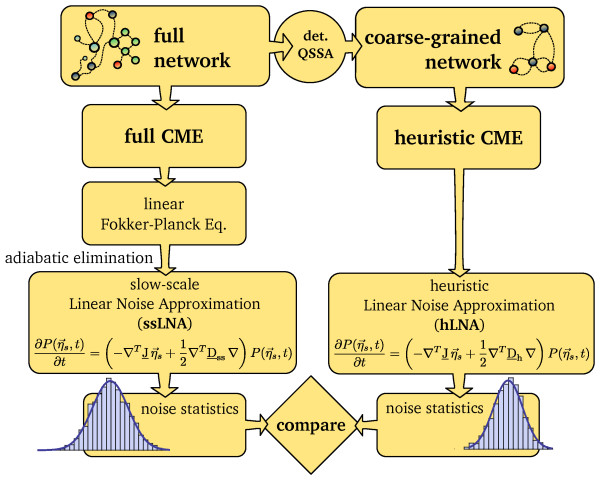
**Determining the validity of the heuristic CME.** Scheme illustrating the analytical approach to determine the validity of the heuristic CME which is used in this article. Parameters are chosen such that the deterministic QSSA is valid and such that molecule numbers are not too small. The LNA is applied to the heuristic CME leading to a linear FPE, describing the noise of the slow species. A different reduced linear FPE describing the noise in the slow species is obtained by applying a rigorous adiabatic elimination method on the linear FPE which approximates the CME of the full network. The noise statistics from the two linear FPEs are compared

The derivation of the ssLNA leads us to a fundamental conclusion: *although the existence of an effective CME for a coarse-grained network under conditions of timescale separation cannot be generally guaranteed (as proved by Janssen), it is always possible to write down a effective linear FPE for the coarse-grained network.* We now also have a viable strategy to compare the heuristic and full CMEs under conditions of timescale separation: one obtains the noise statistics from the hLNA and the ssLNA and compares them for rate constants such that the deterministic QSSA is a valid approximation (for an illustration of the comparison method, see Figure 2). Furthermore, the ssLNA provides us not only with a new method to analytically obtain the noise statistics of a coarse-grained network but also with a new simulation tool which replaces conventional SSA simulations with heuristic propensities. The new simulation method consists in numerically solving the set of stochastic differential equations (Langevin equations) which exactly correspond to equation (9) [37]

(12) $$\frac{d}{\mathrm{dt}}{\vec{\eta }}_{s}(t)=J\;\;{\vec{\eta }}_{s}(t)+\Omega^{-1/2}\left(S{\;}_{s}-J{\;}_{\mathrm{sf}}J\;{\;}_{f}^{-1}S\;{\;}_{f}\right)\sqrt{F}\vec{\Gamma }(t),$$

where the *R* dimensional vector $\vec{\Gamma }(t)$ is white Gaussian noise defined by 〈 Γ_*i*_(*t*)〉=0and $\langle \Gamma_{i}(t)\Gamma_{j}(t^{\prime })\rangle \;=\;\delta_{i,j}\delta (t\;-\;t^{\prime })$.

One may ask whether there is an effective CME which in the large volume limit can be approximated by the ssLNA, Eq. (12). The form of the noise coefficient in Eq. (12) implies that the ssLNA corresponds to the master equation of an effective reaction scheme with a stoichiometric matrix

(13) $$S^{\prime }=S{\;}_{s}-J{\;}_{sf}J\;{\;}_{f}^{-1}S\;{\;}_{f}.$$

Such a reaction scheme is compatible with the reduced REs: defining $\left[{\vec{X}}_{s}\right]$ as the macroscopic concentration vector of the slow species, we have $d[{\vec{X}}_{s}]/dt=S^{\prime }\;\;\vec{f}([{\vec{X}}_{s}])=S{\;}_{s}\vec{f}([{\vec{X}}_{s}])$ since $S\;{\;}_{f}\;\vec{f}=0$ as required by the deterministic QSSA. Note that while S’ is not the only stoichiometric matrix which is compatible with the reduced REs (any matrix of the form $S{\;}_{s}+A\;\;S\;{\;}_{f}$ where A is some general matrix will do), it is the matrix which is uniquely selected by adiabatic elimination of the concentration fluctuations of the fast species. Of course this reduced reaction scheme characterized by S’ is only physically meaningful if its entries are time-independent and integer-valued. Under timescale separation, this condition is not generally fulfilled. Rather this constitutes an additional, stronger condition. Hence it follows that generally a reduced CME description does not exist under timescale separation conditions, though a reduced Langevin equation description, i.e., the ssLNA, always exists.

In the rest of this article, we apply the systematic comparison method developed in the Results section to two examples of biological importance: enzyme-facilitated catalysis of substrate into product by cooperative and non-cooperative mechanisms and a genetic network with a negative feedback loop. For each of these, we shall obtain the noise properties of the coarse-grained versions of the circuits in the limit of large molecule numbers using the ssLNA and the hLNA. Because the expressions for the noise statistics from these two are quite simple, we shall be able to readily identify the regions of parameter space where the hLNA, and hence the heuristic CME, is correct and where it gives misleading results. The theoretical results are confirmed by stochastic simulations based on the CME of the full network and on the heuristic CME of the coarse-grained network.

### Application I: Cooperative and non-cooperative catalytic mechanisms

Many regulatory mechanisms in intracellular biochemistry involve multisubunit enzymes with multiple binding sites [38]. We consider a simple network involving the catalysis of substrate into product by a two- subunit enzyme

(14) $$\begin{array}{lll} & \overset{k_{\mathrm{in}}}{\to }S,\; & \;\\ & S+\mathrm{EE}\overset{k_{1}}{\underset{k_{-1}}{⇌}}\mathrm{EES}\overset{k_{3}}{\to }\mathrm{EE}+P,\; & \;\\ & S+\mathrm{EES}\overset{k_{2}}{\underset{k_{-2}}{⇌}}\mathrm{SEES}\overset{k_{4}}{\to }\mathrm{EES}+\mathrm{P.}\; \end{array}$$

Substrate *S* is input into the compartment where the reaction is occurring, it reversibly binds with an enzyme *EE* which has two free binding sites to form the first complex *EES* with one binding site occupied by a substrate molecule. This complex either decays into the original enzyme *EE* and a product molecule or else it can reversibly bind to another substrate molecule leading to a second complex *SEES* with both binding sites occupied by substrate molecules. Finally, this last complex decays into the first complex and product *P*. Note that reaction scheme (14) is the considered full network, since only elementary reactions are involved.

#### Deterministic analysis and network coarse-graining

The full network (14) (without the input reaction) has been previously studied using REs by Tyson [39]. The coarse-grained network is obtained by implementing the deterministic QSSA: transients in the enzyme and complex concentrations are assumed to decay much faster than transients in the substrate concentrations. Hence, the time derivatives of the REs for the concentrations of the two complexes are set to zero, the steady-state concentrations of the two complexes are found and substituted in the RE for substrate concentration, leading to a single effective rate equation [39] given by

(15) $$\frac{d\;[S]}{\mathrm{dt}}=k_{\mathrm{in}}-k^{\prime }[S],$$

(16) $$k^{\prime }=\frac{\frac{\left[EE_{T}\right]}{K_{m1}}\left(k_{3}+k_{4}\frac{\left[S\right]}{K_{m2}}\right)}{1+\frac{\left[S\right]}{K_{m1}}+\frac{{\left[S\right]}^{2}}{K_{m1}K_{m2}}},$$

where *S* is the instantaneous substrate concentration and *k*^*′*^is an effective first-order rate coefficient. The Michaelis-Menten constants are $K_{m1}=\left(k_{-1}+k_{3}\right)/k_{1}$ and $K_{m2}=\left(k_{-2}+k_{4}\right)/k_{2}$ and the total enzyme concentration is denoted as $\left[EE_{T}\right]$, which is a constant at all times and equal to $\left[EE_{T}\right]=[\mathrm{EE}]+[\mathrm{EES}]+[\mathrm{SEES}]$, the sum of the concentrations of free enzyme and of the two complexed forms. Hence, the coarse-grained version of the full network (14) is simply

(17) $$\begin{array}{ll} & \overset{k_{\mathrm{in}}}{\to }S\overset{k^{\prime }}{\to }\mathrm{P.}\; \end{array}$$

**Figure 3:**
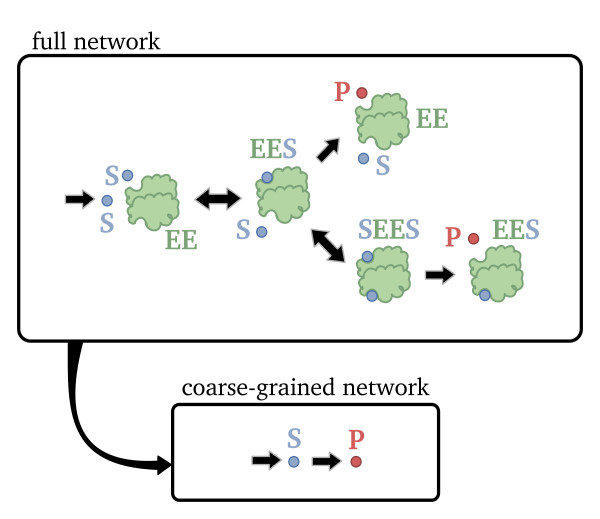
**Full and coarse-grained mechanisms of a two-subunit enzyme network.** Cartoon illustrating the full and coarse-grained networks for the two-subunit enzyme network. The reduced, coarse-grained network is obtained from the full network under conditions of timescale separation, i.e., transients in the concentrations of all enzyme and complex species decay much faster than transients in the concentrations of the substrate and product species

Note that the deterministic QSSA has reduced our network from one with 5 species interacting via 7 elementary reactions, reaction scheme (14), to one with 2 species interacting via 2 reactions, one elementary and one non-elementary, reaction scheme (17). A cartoon representation of the two networks can be found in Figure 3. The dynamics of the coarse-grained network are a good approximation of those of the full network provided the timescales for the decay of the transients in the concentrations of the two complexes are much shorter than those of the substrate.

Note that throughout the rest of this article, the notation [*X*] will generally denote the steady-state concentration of species *X*, unless it appears in the context of a differential equation as in equation (15) where then it necessarily refers to the instantaneous concentration.

#### Stochastic analysis of the coarse-grained network: ssLNA and hLNA methods

We use the ssLNA (see the Results section) to obtain the Langevin equation for the intrinsic noise *η*_*s*_(*t*) about the steady-state macroscopic substrate concentration of the coarse-grained network, i.e., the steady-state solution [*S*] of equation (15). The derivation leading to the Langevin equation can be found in the Methods section. The result is

(18) $$\begin{array}{lll} \;\frac{d}{\mathrm{dt}}\eta_{s}(t) & =J\eta_{s}(t)\;+\;\Omega^{-1/2}\left(\;\sqrt{k_{\mathrm{in}}}\Gamma_{1}(t)\;-\;\sqrt{k_{1}[\mathrm{EE}][S]}q_{1}\;\Gamma_{2}(t)\right)\; & \;\\ & \;+\sqrt{k_{-1}[\mathrm{EES}]}q_{1}\Gamma_{3}(t)\;-\;\sqrt{k_{3}[\mathrm{EES}]}\left(1\;-\;q_{1}\right)\;\Gamma_{4}(t)\; & \;\\ & \;-\sqrt{k_{2}[\mathrm{EES}][S]}q_{2}\Gamma_{5}(t)+\sqrt{k_{-2}[\mathrm{SEES}]}q_{2}\Gamma_{6}(t)\; & \;\\ & \left(\right)\;-\sqrt{k_{4}[\mathrm{SEES}]}\left(1-q_{2}\right)\Gamma_{7}(t)\left(\right)),\; \end{array}$$

where *J* is the Jacobian of the reduced RE, equation (15), and the functions *q*_1_ and *q*_2_ are defined as

(19) $$q_{1}=\frac{k_{3}K_{m2}+k_{4}[S]}{k_{1}\left({\left[S\right]}^{2}+[S]K_{m2}+K_{m1}K_{m2}\right)},$$

(20) $$q_{2}=\frac{k_{4}K_{m1}+\left(k_{4}-k_{3}\right)[S]}{k_{2}\left({\left[S\right]}^{2}+[S]K_{m2}+K_{m1}K_{m2}\right)}.$$

Note that Γ_*i*_(*t*)denotes the contribution to the intrinsic noise in the steady-state substrate concentration due to the *i*th elementary reaction of the full network of which there are 7 in total. It is clear that Γ_1_(*t*) is the noise from the input reaction since it has a pre-factor of *k*_*in*_, Γ_2_(*t*)is the noise from the binding of substrate and free enzyme since it has a pre-factor of *k*_1_ and so on for the rest of the noise terms. Hence, we see that according to the ssLNA, *under conditions of timescale separation, all elementary reactions in the full network contribute to the intrinsic noise in the substrate concentration*. The variance of the intrinsic noise described by the Langevin equation, equation (18), can be calculated according to the recipe described in the Results section (see also the Methods section) and is found to be given by

(21) $$\begin{array}{lll} \;\sigma_{\mathrm{ssLNA}}^{2} & =-{\left(2J\Omega \right)}^{-1}\left(k_{\mathrm{in}}+\left(k_{1}[\mathrm{EE}][S]+k_{-1}[\mathrm{EES}]\right)q_{1}^{2}\right)\; & \;\\ & \;+\left(k_{2}[\mathrm{EES}][S]+k_{-2}[\mathrm{SEES}]\right)q_{2}^{2}+k_{3}[\mathrm{EES}]\; & \;\\ & \;\times \left({\left(1-q_{1}\right)}^{2}+k_{4}[\mathrm{SEES}]{\left(1-q_{2}\right)}^{2}\right).\; \end{array}$$

Next we obtain the variance of the substrate fluctuations by applying the LNA to the heuristic CME of the coarse-grained network (hLNA). The heuristic microscopic rate functions, i.e., the propensities divided by the volume, are in this case

(22) $${\hat{f}}_{1}=k_{\mathrm{in}},$$

(23) $${\hat{f}}_{2}=\frac{n_{S}}{\Omega }k^{\prime }{|}_{\left[S(t)\right]\to n_{s}/\Omega }\;.$$

Using the prescription for the hLNA (see the Results and Methods sections), one obtains the variance of the fluctuations to be

(24) $$\begin{array}{ll} \sigma_{\mathrm{hLNA}}^{2}=-{\left(2J\Omega \right)}^{-1}(k_{\mathrm{in}}+k_{3}[\mathrm{EES}]+k_{4}[\mathrm{SEES}]). & \; \end{array}$$

A comparison of equations (21) and (24) leads one to the observation that the latter can be obtained from the former by setting *q*_1_=*q*_2_=0. Substituting these conditions in the Langevin equation, equation (18), we obtain physical insight into the shortcomings of the conventional heuristic method. This method rests on the incorrect implicit assumption that *under conditions of timescale separation, the reversible elementary reactions involving the fast species do not contribute to the intrinsic noise in the substrate concentration*.

#### Stochastic Michaelis-Menten and Hill-type kinetics

We now consider two subcases which are of special interest in biochemical kinetics: (i) *k*_2_→0, *K*_*m*2_→*∞*; (ii) *k*_2_→*∞*, *K*_*m*2_→0 at constant $K_{m1}K_{m2}=K_{m}^{2}$. These limits applied to the reduced RE, equations (15), lead to the two simplified REs, respectively,

(25) $$\text{Case (i)}\;\;\frac{d[S]}{\mathrm{dt}}=k_{\mathrm{in}}-\frac{k_{3}\left[EE_{T}\right][S]}{K_{m1}+[S]},$$

(26) $$\text{Case (ii)}\;\;\frac{d[S]}{\mathrm{dt}}=k_{\mathrm{in}}-\frac{k_{4}\left[EE_{T}\right]{\left[S\right]}^{2}}{K_{m}^{2}+{\left[S\right]}^{2}}.$$

Hence, the first case leads to Michaelis-Menten (MM) kinetics (non-cooperative kinetics) and the second to Hill-type kinetics with a Hill coefficient of two (cooperative kinetics).

Applying limit (i) to equations (21) and (24), we obtain the variance of the fluctuations for Michaelis-Menten kinetics as predicted by the ssLNA and the hLNA

(27) $$\text{MM-kinetics}\;\;\sigma_{\mathrm{ssLNA}}^{2}=\frac{\left[S\right]}{\Omega }\left(1+\frac{k_{-1}/k_{1}+[S]}{K_{m1}+[S]}\;\frac{\left[S\right]}{K_{m1}}\right)\;,$$

(28) $$\sigma_{\mathrm{hLNA}}^{2}=\frac{\left[S\right]}{\Omega }\left(1+\frac{\left[S\right]}{K_{m1}}\right).$$

Similarly applying limit (ii) to equations (21) and (24), we obtain the variance of the fluctuations for Hill-type kinetics as predicted by the ssLNA and the hLNA

(29) $$\;\text{Hill-kinetics}\;\sigma_{\mathrm{ssLNA}}^{2}=\frac{\left[S\right]}{2\Omega }\frac{K_{m}^{2}+{\left[S\right]}^{2}}{K_{m}^{2}}\;+\;\frac{k_{4}}{2k_{1}\Omega }\frac{{\left[S\right]}^{2}}{{\left[S\right]}^{2}+K_{m}^{2}},$$

(30) $$\sigma_{\mathrm{hLNA}}^{2}=\frac{\left[S\right]}{2\Omega }\frac{K_{m}^{2}+{\left[S\right]}^{2}}{K_{m}^{2}}.$$

Comparison of equations (27) and (28) shows that the *heuristic CME description of the coarse-grained network overestimates the size of intrinsic noise whenever the deterministic kinetics are Michaelis-Menten*. Interestingly, comparison of equations (29) and (30) shows the opposite *for Hill-type kinetics: the size of noise predicted by the heuristic CME underestimates the true value*. Note also that for both types of kinetics, the heuristic CME predicts the correct noise statistics in the limit of very small and very large substrate concentrations (which correspond to very large and very small free enzyme concentrations in steady-state conditions, respectively). The predictions for the Michaelis-Menten case agree with those reported by a recent simulation-based study [17] and a study using the LNA applied to the full network [9]. Indeed, this agreement is an important benchmark for the ssLNA. To our knowledge, the results for the Hill-type kinetics have not been obtained before.

**Figure 4:**
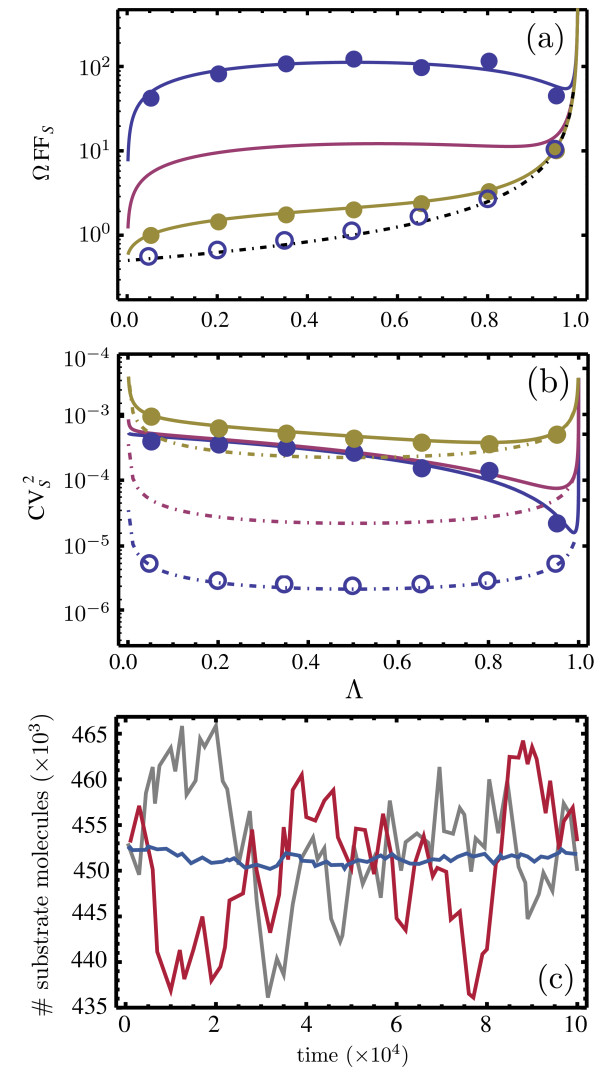
**Noise statistics for cooperative two-subunit enzyme network.** Plots showing the Fano factor multiplied by the volume, ΩFF_S_, (**a**) and the coefficient of variation squared, ${\text{CV}}_{\text{S}}^{2}$, (**b**) for the substrate fluctuations as a function of the non-dimensional fraction *Θ*, in steady-state conditions. The latter is a measure of enzyme saturation. The solid lines are the ssLNA predictions, the dashed lines are the hLNA predictions, the solid circles are obtained from stochastic simulations of the full network and the open circles are obtained from stochastic simulations of the coarse-grained network using the SSA with heuristic propensities. The color coding indicates different values of the bimolecular rate constant *k*_1_: 5×10^−3^(yellow), 5×10^−5^(purple), and 5×10^−7^(blue). The remaining parameters are given by $\left[EE_{T}\right]=1$, *k*_2_=1000, *k*_−1_=*k*_−2_=100, *k*_3_=*k*_4_=1. Note that in (a) the black dashed line indicates the hLNA prediction for all three different values of *k*_1_, which are indistinguishable in this figure. The stochastic simulations were carried out for a volume Ω=100. In (**c**) sample paths of the SSA for the full network (gray), the slow scale Langevin equation (red) as given by equation (18) and the SSA with heuristic propensities (blue) are compared for *Θ*=0.5. The slow scale Langevin equation is numerically solved using the Euler-Maruyama method with timestep *δt*=0.1. Note that in all cases, the chosen parameters guarantee timescale separation (validity of the deterministic QSSA) (see Figure 5)

**Figure 5:**
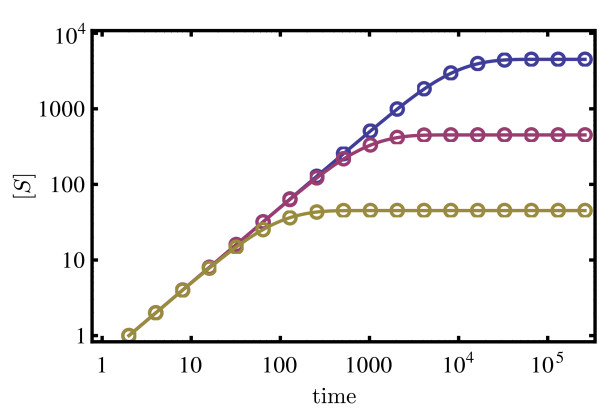
**Validity of the deterministic QSSA for the cooperative two-subunit enzyme network.** Plot of the macroscopic substrate concentration [*S*] versus time, as obtained by numerically solving the deterministic REs of the full network (solid lines) and the reduced REs obtained using the deterministic QSSA (open circles). The color coding and rate constant values are as in Figure 4 (a) and (b); the value of *Θ*is 0.5. The excellent agreement between the two RE solutions, implies timescale separation conditions

The results for Hill-type kinetics are shown in Figure 4. In Figures 4a and 4b we plot the coefficient of variation CV_S_ and the Fano factor FF_S_ of the substrate concentration fluctuations (as predicted by equations (29) and (30)) versus the non-dimensional fraction $\Theta =k_{\mathrm{in}}/k_{4}\left[EE_{T}\right]$. From equation (26) it can be deduced that ${\left[S\right]}^{2}=K_{m}^{2}\Theta /(1-\Theta )$; hence, the physical meaning of *Θ*is that it is a measure of enzyme saturation since as it increases, the substrate concentration increases as well, and consequently the free enzyme concentration decreases. The values of rate constants are chosen such that timescale separation is guaranteed, i.e., there is very good agreement between the concentration of the slow species as predicted by the REs of the full network and the reduced REs obtained using the deterministic QSSA (see Figure 5).

The following observations can be made from Figures 4a and 4b. The ssLNA quantitatively agrees with the results of stochastic simulations of the full network for a large enough volume Ω. In contrast, the heuristic approach, hLNA, and stochastic simulations based on the corresponding heuristic CME, are generally in quantitative disagreement with the results of the ssLNA and of stochastic simulations of the full network, even if the volume is very large. For example, for the case *k*_1_=5×10^−7^ and *Θ*=1/2, the CV_S_ and FF_S_ from the hLNA are approximately 11 and 112 times smaller, respectively, than the prediction of the ssLNA. In Figure 4c we also illustrate the large differences which these statistics imply, by showing sample paths (trajectories) of the CME of the full network, of the heuristic CME and of the Langevin equation given by the ssLNA, equation (18). This confirms that: (i) the hLNA and, hence, the heuristic CME on which it is based, predicts the correct mean concentrations but incorrect noise statistics even if the molecule numbers are considerably large; (ii) the Langevin equation obtained from the ssLNA is a viable accurate simulation alternative to SSA simulations based on the heuristic CME.

Besides quantitative disagreement we also note that the qualitative dependence of the FF_S_ and the CV_S_ with *Θ* as predicted by the heuristic approach is also very different than the predictions of the ssLNA and stochastic simulations with the full network. For example, for the case *k*_1_=5×10^−7^, according to stochastic simulations of the full network and the ssLNA, the FF_S_ reaches a maximum at *Θ*=1/2, whereas the heuristic approach predicts a monotonic increase of the FF_S_with *Θ*. The case *Θ*<0.5is particularly interesting because the ssLNA and stochastic simulations of the full network lead to ΩFF_S_which is much greater than 1, whereas the heuristic approach predicts ΩFF_S_ which is below 1. Hence, for *Θ*<0.5, the ssLNA correctly predicts the fluctuations to be super-Poissonian, i.e., the size of the fluctuations is larger than those of a Poissonian with the same mean number of substrate molecules, whereas the hLNA incorrectly predicts the opposite case of sub-Poissonian fluctuations.

The power spectrum for the substrate fluctuations has also been calculated (see the Methods section). Although there is some quantitative disagreement between the predictions of the ssLNA and hLNA both are in qualitative agreement: the spectrum is monotonic in the frequency and hence no noise-induced oscillations are possible by this mechanism. More generally, it can be shown that the spectra of the hLNA and ssLNA are in qualitative agreement for all full networks with at most one slow species because as can be deduced from equation (7), for such networks, the spectrum for a single species chemical system is invariably monotonic in the frequency.

### Application II: A gene network with negative feedback

Finally, we study an example of a gene network with autoregulatory negative feedback. Such a feedback mechanism is ubiquitous in biology appearing in such diverse contexts as metabolism [40], signaling [41], somitogenesis [42] and circadian clocks [43]. Two reasons hypothesized for its widespread occurance are that (i) it supresses the size of intrinsic noise [44,45] thereby providing enhanced stability and (ii) it can lead to concentration oscillations or rhythms which are crucial to the control of several aspects of cell physiology [36].

We consider the following prototypical gene network. For convenience, we divide the network into two parts: (i) the set of reactions which describe transcription, translation and degradation, and (ii) the set of reactions which constitute the negative feedback loop. The first part is described by the reactions

(31) $$\begin{array}{lll} & G\overset{k_{0}}{\to }G+M,\; & \;\\ & M\overset{k_{s}}{\to }P+M,\; & \;\\ & P+E\overset{k_{3}}{\underset{k_{-3}}{⇌}}\mathrm{EP}\overset{k_{4}}{\to }E,\; & \;\\ & M\overset{k_{\mathrm{dM}}}{\to }\emptyset .\; \end{array}$$

The mRNA, *M*, is produced by transcription from a single gene *G*, and it is translated into protein *P* which subsequently is degraded via an enzymatic reaction catalyzed by enzyme *E*. The mRNA can furthermore decay into an inactive form spontaneously. In addition, we have a negative feedback loop described by the reactions

(32) $$\begin{array}{lll} & P+G\overset{k_{1}}{\underset{k_{-1}}{⇌}}\mathrm{GP},\; & \;\\ & P+\mathrm{GP}\overset{k_{2}}{\underset{k_{-2}}{⇌}}GP_{2},\; & \;\\ & \mathrm{GP}\overset{k_{0}}{\to }\mathrm{GP}+\mathrm{M.}\; \end{array}$$

Note that the gene with two bound proteins is inactive, in the sense that it does not lead to mRNA production. This implies that sudden increases in protein concentration lead to a decrease in mRNA transcription which eventually results in a lowered protein concentration; this is the negative feedback or auto-inhibitory mechanism. The reaction network as given by reaction schemes (31) and (32) is our full network for this example. Note that the first two reactions in reaction scheme (31) are not in reality elementary chemical reactions but they are the simplest accepted forms of modeling the complex processes of transcription and translation and hence it is in this spirit that we include them in our full network description.

#### Deterministic analysis and coarse-grained network

**Figure 6:**
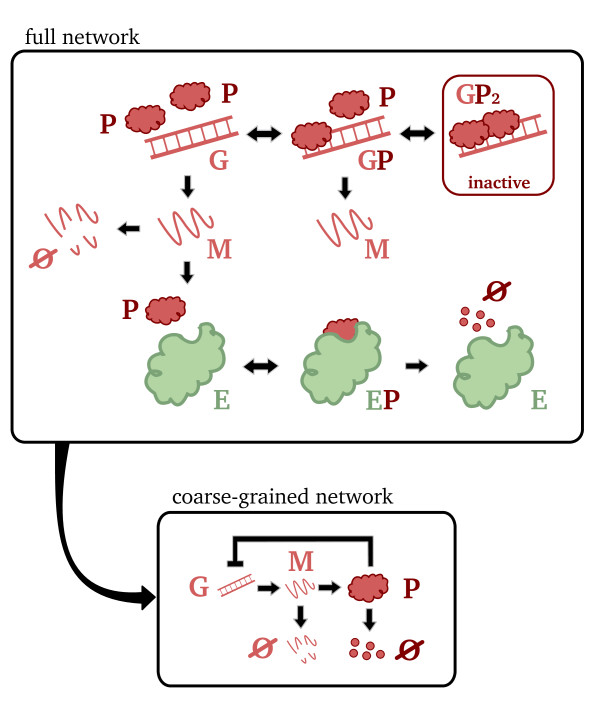
**Full and coarse-grained mechanisms of a gene network.** Cartoon illustrating the full and coarse-grained networks for the gene network with a single negative feedback loop. The reduced, coarse-grained network is obtained from the full network under conditions of timescale separation, i.e., transients in the concentrations of all enzyme, enzyme complex, gene and gene complex species decay much faster than transients in the concentrations of mRNA and protein

Model reduction on the macroscopic level proceeds by applying the deterministic QSSA to the REs of the full network (see the Methods section for details). The fast species are the enzyme, *E*, the enzyme complex, *EP*, and the gene species in its various non-complexed and complexed forms *G*, *GP* and *GP*_2_. The slow species are the mRNA, *M*, and the protein, *P*. Furthermore, we also impose the limit *k*_2_→*∞*, *k*_1_→0 at constant *k*_2_*k*_1_; this enforces cooperative behavior since the binding of *P* to *G* is quite slow but once it occurs the next binding of *P* to the complex *GP* is very quick. The resultant reduced REs are given by

(33) $$\begin{array}{lll} \frac{d\;[M]}{\mathrm{dt}} & =\frac{k_{0}\left[G_{T}\right]K^{2}}{K^{2}+{\left[P\right]}^{2}}-k_{\mathrm{dM}}[M],\; & \;\\ \frac{d\;[P]}{\mathrm{dt}} & =k_{s}[M]-\frac{k_{4}\left[E_{T}\right][P]}{K_{M}+[P]},\; \end{array}$$

where *K*^2^=*k*_−1_*k*_−2_/*k*_1_*k*_2_, $K_{M}=\left(k_{-3}+k_{4}\right)/k_{3}$, $\left[E_{T}\right]$ is the total enzyme concentration and $\left[G_{T}\right]$ is the total gene concentration. The model reduction process just described is illustrated in Figure 6.

#### Stochastic analysis of the coarse-grained network: ssLNA and hLNA methods

We denote *η*_*s*,1_and *η*_*s*,2_ as the fluctuations about the concentrations of mRNA and of protein, respectively. The ssLNA leads to reduced Langevin equations of the form

(34) $$\begin{array}{lll} \frac{d}{\mathrm{dt}}\eta_{s,1}(t) & =J_{11}\eta_{s,1}(t)+J_{12}\eta_{s,2}(t)+\Omega^{-1/2}\; & \;\\ & \;\times \left(\sqrt{k_{0}[G]}\Gamma_{1}(t)+\sqrt{k_{0}[\mathrm{GP}]}\Gamma_{2}(t)\right)\; & \;\\ & \;-p_{1}\sqrt{k_{1}[G][P]}\Gamma_{3}(t)\;+\;p_{1}\sqrt{k_{-1}[\mathrm{GP}]}\Gamma_{4}(t)\; & \;\\ & \left(\;-\sqrt{k_{\mathrm{dM}}[M]}\Gamma_{7}(t)\right),\; & \;\\ \frac{d}{\mathrm{dt}}\eta_{s,2}(t) & =J_{21}\eta_{s,1}(t)+J_{22}\eta_{s,2}(t)+\Omega^{-1/2}\; & \;\\ & \;\times \left(\sqrt{k_{s}[M]}\Gamma_{8}(t)\;-\;(1\;-\;q)\sqrt{k_{3}[E][P]}\Gamma_{9}(t)\right)\; & \;\\ & \;+(1-q)\sqrt{k_{-3}[\mathrm{EP}]}\Gamma_{10}(t)\; & \;\\ & \left(\;-q\sqrt{k_{4}[\mathrm{EP}]}\Gamma_{11}(t)\right),\; \end{array}$$

where Γ_*i*_(*t*) is the noise contributed by reaction number *i* and the reactions are numbered according to the order: *G*→*G* + *M*, *GP*→*GP* + *M*, *P* + *G*→*GP*, *GP*→*P* + *G*, *P* + *GP*→*GP*_2_, *GP*_2_→*P* + *GP*, *M*→∅, *M*→*M* + *P*, *P* + *E*→*EP*, *EP*→*P* + *E*, and *EP*→*E*. The element *J*_*ij*_ denotes the *i*-*j*entry of the Jacobian J of the reduced REs (33). Furthermore, the parameters *p*_1_ and *q* are given by

(35) $$p_{1}=\frac{k_{0}}{k_{1}}\frac{\left[P\right]}{K^{2}+{\left[P\right]}^{2}},$$

(36) $$q=\frac{[P]+K_{3}}{[P]+K_{M}},$$

where *K*_3_=*k*_−3_/*k*_3_. Note that the coupled Langevin equations (34) imply that the fluctuations in the mRNA and protein concentrations are affected by noise from all of the 11 constituent reactions of the full network (reaction schemes (31) and (32)) except from those of the reversible reaction *P* + *GP⇌GP*_2_. As shown in the Methods section, the noise from this reaction becomes zero due to the imposition of cooperative behavior in the feedback loop.

The covariance matrix for the fluctuations of the Langevin equations (34) is given by the Lyapunov equation (6) with Jacobian being equal to that of the reduced REs (33) and diffusion matrix D_*h*_ replaced by D_*ss*_, which is given by

(37) $$\begin{array}{lll} D{\;}_{\mathrm{ss}} & =\Omega^{-1}\text{diag}\left(D_{M},D_{P}\right),\; & \;\\ D_{M} & =k_{\mathrm{dM}}[M]+\frac{k_{0}\left[G_{T}\right]K^{2}}{K^{2}+{\left[P\right]}^{2}}+p_{1}\frac{2k_{0}\left[G_{T}\right]K^{2}{\left[P\right]}^{2}}{{\left(K^{2}+{\left[P\right]}^{2}\right)}^{2}},\; & \;\\ D_{P} & =k_{s}[M]+\frac{k_{4}\left[E_{T}\right][P]}{K_{M}+[P]}-(1-q)\frac{2k_{4}\left[E_{T}\right]{\left[P\right]}^{2}}{{\left(K_{M}+[P]\right)}^{2}}.\; \end{array}$$

It is also possible to calculate the covariance matrix of the fluctuations of the slow variables using the hLNA (see the Methods section). This is given by a Lyapunov equation (6) with Jacobian being equal to that of the reduced REs (33) and diffusion matrix D_*h*_ given by

(38) $$\begin{array}{lll} D{\;}_{h} & =\Omega^{-1}\text{diag}\left(D_{h,M},D_{h,P}\right),\; & \;\\ D_{h,M} & =k_{\mathrm{dM}}[M]+\frac{k_{0}\left[G_{T}\right]K^{2}}{K^{2}+{\left[P\right]}^{2}},\; & \;\\ D_{h,P} & =k_{s}[M]+\frac{k_{4}\left[E_{T}\right][P]}{K_{M}+[P]}.\; \end{array}$$

A comparison of equation (37) and equation (38) shows that the ssLNA and hLNA are generally different except in the limits of *p*_1_→0 and *q*→1. From the Langevin equations (34) we see that setting *p*_1_=0 implies ignoring the noise due to the reversible reaction *P* + *G⇌GP*, while setting *q*=1 is equivalent to ignoring the noise from the reversible reaction *P* + *E⇌EP*. Hence, as for the previous example of enzyme kinetics, we can state that the hLNA and the heuristic CME upon which it rests, implicitly (and incorrectly) assume that *under conditions of timescale separation, the reversible reactions involving the fast species do not contribute to the intrinsic noise in the slow species*.

Furthermore, by the comparison of equations (37) and (38), one can also deduce that the heuristic CME provides a statistically correct description when the protein concentration [*P*] is either very small or very large, in other words the case of very weak or very strong transcriptional repression (and corresponding non-saturated and saturated degrading enzyme conditions).

#### Detailed comparison of the noise statistics from the ssLNA and hLNA

**Figure 7:**
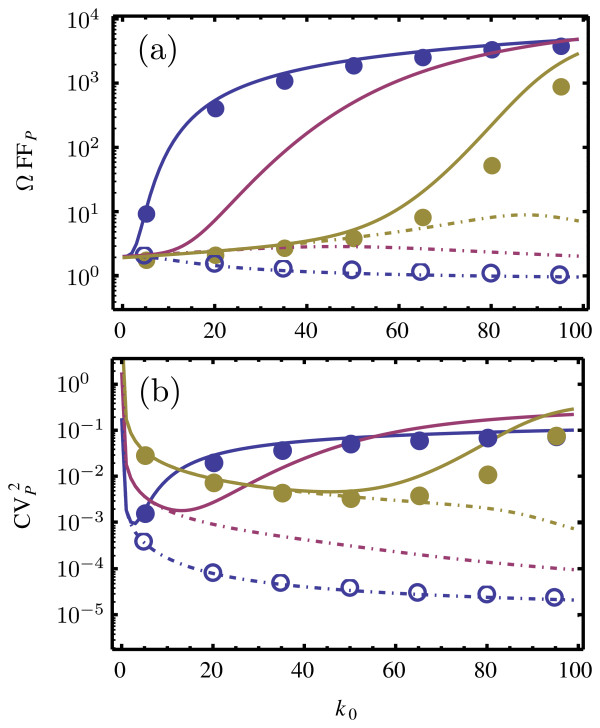
**Noise statistics of the gene network.** Dependence of the Fano factor (**a**) and of the coefficient of variation squared (**b**) of the protein fluctuations on the rate of transcription *k*_0_, according to the ssLNA (solid lines) and the hLNA (dashed lines). The noise measures are calculated for three values of the bimolecular constant *k*_3_=1(yellow), *k*_3_=0.1(purple), *k*_3_=0.01(blue). All other parameters are given by $\left[G_{T}\right]=0.01$, $\left[E_{T}\right]=1$, *k*_1_=10^−5^, *k*_2_=100, *k*_−1_=*k*_−2_=*k*_−3_=10, *k*_4_=*k*_*s*_=*k*_*dM*_=1. Stochastic simulations of the full networks (solid circles) and of the coarse-grained network (open circles) using the CME and the heuristic CME, respectively, were performed for a volume of Ω=100. Note that at this volume there is one gene and 100 enzyme molecules. Note also that the chosen parameters guarantee timescale separation (validity of the deterministic QSSA) and cooperative behavior in the feedback loop (see Figure 8)

**Figure 8:**
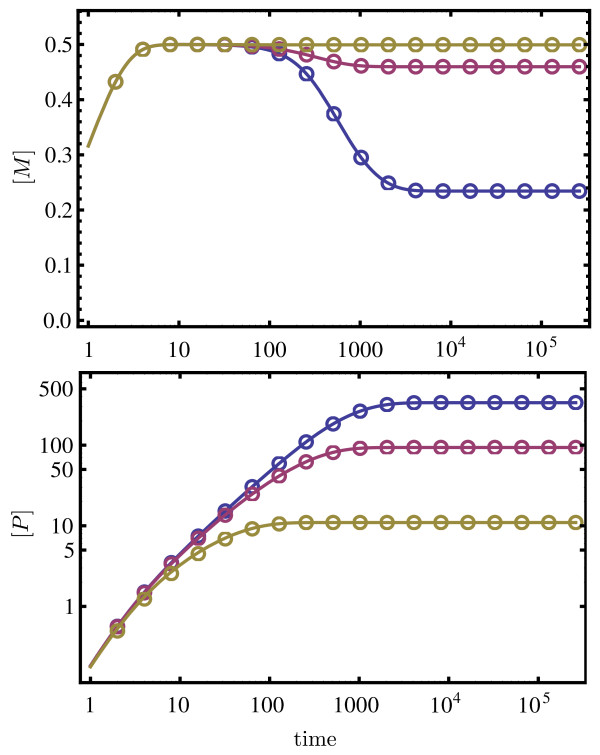
**Validity of the deterministic QSSA for the gene network.** Plot of the macroscopic substrate concentrations of mRNA, [*M*], and protein, [*P*], versus time, as obtained by numerically solving the deterministic REs of the full network (solid lines) and the reduced REs obtained using the deterministic QSSA (open circles). The color coding and rate constant values are as in Figure 7; the value of *k*_0_is 50. The excellent agreement between the two RE solutions, implies timescale separation conditions

Figure 7 shows the ssLNA and hLNA predictions for the coefficient of variation squared and the Fano factor of the protein fluctuations as a function of the transcription rate *k*_0_. These are obtained by solving the two Lyapunov equations mentioned in the previous subsection for the covariance matrix; the variances are then the diagonal elements of this matrix, from which one finally calculates the Fano factors and the coefficients of variation. The values of rate constants are chosen such that we have timescale separation conditions (see Figure 8). From Figure 7 we can see that under such conditions, the ssLNA predictions agree very well with the stochastic simulations of the full network but the hLNA exhibits considerable quantitative and qualitative differences compared to the latter simulations. In particular, note that for *k*_3_=1 and *k*_0_>50, the predictions of the ssLNA are approximately 3 orders of magnitude larger than those of the hLNA (and of stochastic simulations using the heuristic CME).

**Figure 9:**
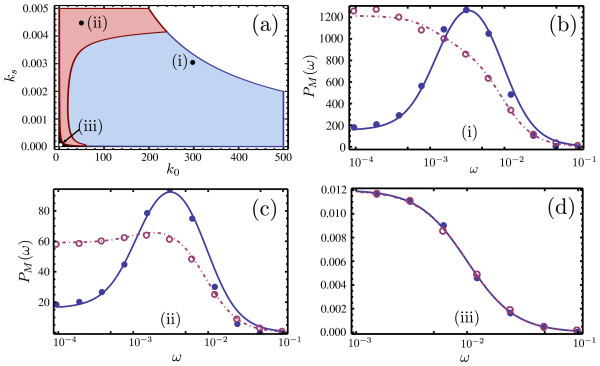
**Noise-induced oscillations in the gene network.** Comparison of the predictions of noise-induced oscillations in the mRNA concentrations by ssLNA and hLNA methods. Panel (**a**) shows a stochastic bifurcation diagram depicting the regions in the translation rate (*k*_*s*_) versus transcription rate (*k*_0_) parameter space where both methods predict no oscillations (black), both predict oscillations (red) and only the ssLNA correctly predicts an oscillation (blue). There is no steady-state in the white region. Panels (**b**), (**c**) and (**d**) show spectra at 3 points in the blue, red and black regions of the bifurcation plot in **(a)** (these points are marked by roman numbers). The solid and dashed lines show the predictions of the ssLNA and the hLNA respectively, while the dots and circles show the results of stochastic simulations of the full and coarse-grained network using the CME and the heuristic CME, respectively. The parameters are given by Ω=1000, $\left[E_{T}\right]=0.01$, $\left[G_{T}\right]=1/\Omega$ and *k*_dM_=0.01, *k*_1_=0.001, *k*_−1_=100, *k*_2_=1000, *k*_−2_=1, *k*_−3_=10, *k*_3_=0.1, *k*_4_=10. These parameters guarantee timescale separation (validity of the deterministic QSSA) and cooperative behavior in the feedback loop. Note that the hLNA spectrum in (**b**) and (**c**) is scaled up 5000 and 1000 times, respectively

Finally, we investigate the differences between the predictions of the ssLNA and hLNA for noise-induced oscillations in the mRNA concentrations. These are oscillations which are predicted by CME based approaches but not captured by RE approaches. In particular, these noise-induced oscillations occur in regions of parameter space where the REs predict a stable steady-state [46]. Calculation of the power spectra is key to the detection of these noise-induced oscillations: a peak in the spectrum indicates a noise-induced oscillation. For the hLNA this is given by equation (7) with Jacobian being equal to that of the reduced REs, equation (33), and diffusion matrix D_*h*_ given by equation (38). For the ssLNA this is given by equation (7) with Jacobian being equal to that of the reduced REs, equation (33), and diffusion matrix D_*h*_ replaced by D_*ss*_, which is given by equation (37). Since the two diffusion matrices D_*h*_ and D_*ss*_ are not generally equal to each other we expect the spectra calculated according to the ssLNA and hLNA to differ. Indeed we find 3 possible scenarios: both spectra do not have a peak in frequency (no noise-induced oscillations), both spectra have a peak in frequency (noise-induced oscillations) and the most interesting case where the ssLNA spectrum exhibits a peak but the hLNA does not predict one. The results are summarized in Figure 9, where we show the regions of parameter space in which each of these scenarios occur and a comparison of the power spectra as predicted by the hLNA and ssLNA in these regions. Note that in all cases the hLNA (purple dashed lines) agrees with stochastic simulations of the coarse-grained network using the heuristic CME (purple open circles), while the ssLNA (solid blue lines) agrees with stochastic simulations of the full network (blue solid circles) under conditions of timescale separation. The hLNA and ssLNA spectra are only in good quantitative agreement in a very small region of parameter space (shown in black in Figure 9a), where both do predict noise-induced oscillations.

We emphasize that the main message brought by our analysis is that there are significantly large regions of parameter space (blue region in Figure 9a), where the hLNA (and the heuristic CME) does not predict noise-induced oscillations but such oscillations are obtained from stochastic simulations of the full network as well as being captured by the ssLNA.

Qualitative discrepancies in the prediction of noise-induced oscillations arise because the hLNA does not correctly take into account the fluctuations stemming from the rate limiting step of the cooperative binding mechanism. The latter involves the slow binding reaction between a protein molecule *P* and a gene *G* leading to a complex *GP*. The reason for this is the hLNA’s tacit assumption that the fast species are not involved in slow reactions. This rate limiting reaction is at the heart of the negative feedback loop that is responsible for concentration oscillations in many biological networks such as circadian clocks [36] and hence why we speculate that the hLNA misses the occurrence of noise-induced oscillations in certain regions of parameter space.

## Discussion and conclusion

Concluding, in this article we have rigorously derived in closed-form, linear Langevin equations which describe the noise statistics of the fluctuations about the deterministic concentrations as predicted by the reduced REs obtained from the deterministic QSSA. Equivalently, the ssLNA, as the method was called, is the statistically correct description of biochemical networks under conditions of timescale separation and sufficiently large molecule numbers. We note that our method provides an accurate means of performing stochastic simulation in such conditions. This is particularly relevant since it has been proven that there is generally no reduced CME description in such cases [8]. Another advantage of the ssLNA is that it enables quick computation of noise statistics through the solution of a set of simultaneous, deterministic linear algebraic equations. By applying the ssLNA to two biologically relevant networks, we showed that this procedure can lead to particularly simple and compact expressions for the noise statistics, which are in very good numerical agreement with stochastic simulations of the CME of the full network under the above conditions. This is in contrast to the heuristic CME, which generally performed with poor accuracy and in some instances even missed the prediction of noise-induced oscillations.

The limitations of the ssLNA are precisely those of the conventional LNA on which it is based. Namely, if the system is composed of at least one bimolecular reaction, then it is valid for large enough molecule numbers (or, equivalently, large volumes) and provided the biochemical network is monostable. If the system is purely composed of first-order reactions and if one is only interested in variance and power spectra, then the only requirement is that of monostability. This is since in such a case it is well known that the first and second moments are exactly given by the LNA. For monostable systems with bimolecular reactions, the finite-volume corrections to the LNA can be considerable when the network has implicit conservation laws, when bursty phenomena are at play and when steady-states are characterized by few tens or hundreds of molecules [24,47-49]. These problems probably become exacerbated when the network is bistable or possesses absorbing states [50]. Hence, it is clear that although the ssLNA presented in this article is valid for a considerable number of biologically interesting cases, it cannot be homogeneously applied to all intracellular reaction networks of interest. These require the development of methods beyond those presented in this article and hence present an interesting research challenge for the future.

A necessary and sufficient condition for timescale separation is that the timescales governing the decay of the transients in the average concentrations are well separated. Fast species are those whose transients decay on fast timescales while the slow species are those whose transients decay on slow timescales. At the microscopic level, there are several different scenarios which can lead to timescale separation. Grouping chemical reactions as fast or slow according to the relative size of their associated timescales, Pahlajani *et al.*[51] obtain timescale separation by defining fast species as those which are involved in fast reactions only and slow species as those involved in slow reactions only. Zeron and Santillan [52] use a similar but less restrictive approach whereby the fast species are involved in fast reactions only and the slow species can participate in both fast and slow reactions. Another method is that due to Cao *et al.*[53] who define slow species as those involved in slow reactions only and fast species as those participating in at least one fast reaction and any number of slow reactions. While the three aforementioned scenarios will lead to timescale separation, it must be emphasized that they only constitute a subset of the possible scenarios leading to such conditions. The derivation behind the ssLNA is not based on any particular microscopic scenario, rather it simply requires that the timescales of the transients in the average macroscopic concentrations are well separated. Hence it follows that in the limit of large molecule numbers, the methods developed in [51-53] cover only a sub-space of the parameter space over which timescale separation is valid. In the Methods section we indeed show that Pahlajani’s approach [51] leads to a reduced linear Langevin equation which is a special case of the ssLNA, the case where the matrix B in equation (10) can be neglected. The approaches of Zeron and Santillan [52] and of Cao *et al.*[53] lead to a reduced CME description. As we have shown in the Results section, under conditions of timescale separation and for small intrinsic noise, there always exists a reduced linear Langevin description of monostable stochastic reaction networks (the ssLNA) but there is generally not a physically meaningful reduced master equation description. The latter is only obtained if one imposes stronger conditions.

These results are in line with those of Mastny *et al.*[54] which show that for the Michaelis-Menten reaction without substrate input, the sQSPA method, a rigorous singular-perturbation approach, leads to a reduced master equation whenever the free enzyme or complex concentrations are very small (see Table II of Ref. [54]). This equation has the same form as the heuristic CME. This implies that for such conditions the error in the predictions of the heuristic CME should be zero, a result which is reproduced by the ssLNA (see Application I in the Results section). However, note that though these concentration conditions can be compatible with the deterministic QSSA they are not synonymous with it. Generally, the sQSPA methods do not lead to a reduced stochastic description consistent with the deterministic QSSA over the whole parameter space, whereas the ssLNA does, albeit within the constraints that molecule numbers should not be too small and that the network is monostable.

Finally we consider the approach of Shahrezaei and Swain [55], who derived the probability distribution for a linear two-stage model of gene expression under conditions of timescale separation. Their method rests on an exact solution of the generating function equation corresponding to the CME in the limit that the protein lifetime is much greater than that of the mRNA. In the Methods section we show that the ssLNA applied to their example leads to the same variance as obtained from their reduced probability distribution. The upside of their method over the ssLNA is that they obtain the full probability distribution valid for all molecule numbers. The downside of their method is that the generating function equation can only be solved exactly for networks composed of first-order processes (as in the gene example presented by Shahrezaei and Swain) or very simple bimolecular reactions [19] and hence the method has a restricted range of applicability compared to the ssLNA.

While the stochastic simulation algorithm explicitly simulates every individual reaction event, the Langevin approach yields approximate stochastic differential equations for the molecular populations. This is computationally advantageous whenever the reactant populations are quite large [5]. This reasoning can be deduced from the relationship between the propensities and the microscopic rate functions as given by $a_{j}=\Omega {\hat{f}}_{j}(\vec{n}/\Omega )$. It is well known that in the large population number limit, the vector $\vec{n}/\Omega$ is approximately equal to the vector of macroscopic concentrations and hence the magnitude of the propensities increases with the reaction volume or equivalently with molecule numbers. In particular, this implies that the time between consecutive reaction events, given by $\tau =-{\left(\underset{j}{\sum }a_{j}\right)}^{-1}\ln(r)$ where *r*∈(0,1) is a uniform random number, decreases with increasing reaction volume. This means that the time spent by the stochastic simulation algorithm increases with increasing volume because more reaction events have to be resolved within the same time window. Given this reasoning we can compare the discussed methods in terms of speed and accuracy. The computation time of the Langevin methods, hLNA and ssLNA, is independent of the volume and hence if the molecule numbers are not too small, both methods are much quicker than simulating any reduced CME of the coarse-grained network or the CME of the full network. However the ssLNA enjoys the additional advantage that under conditions of timescale separation, it is as accurate as the CME of the full network. The same argument does not generally hold for the hLNA.

We emphasize that besides deriving the ssLNA method, in this paper we have used it to determine the range of validity of the conventional heuristic CME approach and the size of errors in its predictions. To our knowledge, this is the first study which attempts to answer these important and timely questions via a rigorous, systematic theoretical approach.

Our main message is that, the “conventional wisdom” that the heuristic CME is generally a good approximation to the CME of the full network under conditions of timescale separation is incorrect, if one is interested in intrinsic noise statistics and the prediction of noise-induced oscillations.

## Methods

### Derivation of the ssLNA

The linear FPE describing the full network is given by equation (8). It is well known that with every FPE one can associate a set of Langevin equations (stochastic differential equations) [37]. Note that the Langevin and FPE formalisms are exactly equivalent but as we show now, the Langevin description is ideal for deriving a reduced description in timescale separation conditions.

The set of coupled Langevin equations equivalent to equation (8) are

(39) $$\frac{d}{\mathrm{dt}}{\vec{\eta }}_{f}=J\;{\;}_{f}(t){\vec{\eta }}_{f}+J\;{\;}_{\mathrm{fs}}(t){\vec{\eta }}_{s}+\frac{1}{\sqrt{\Omega }}S\;{\;}_{f}\sqrt{F}\;\;\vec{\Gamma }(t),$$

(40) $$\frac{d}{\mathrm{dt}}{\vec{\eta }}_{s}=J{\;}_{\mathrm{sf}}(t){\vec{\eta }}_{f}+J{\;}_{s}(t){\vec{\eta }}_{s}+\frac{1}{\sqrt{\Omega }}S{\;}_{s}\sqrt{F}\;\;\vec{\Gamma }(t).$$

Note that the time-dependence of the matrices in the above equations comes from that of the macroscopic concentrations of fast and slow species. Now say that we impose timescale separation conditions, i.e., the correlation time of fast fluctuations, *τ*_*f*_, is much smaller than the correlation time of slow fluctuations, *τ*_*s*_. We wish to obtain a reduced description for the fast fluctuations, i.e., for equation (39), on timescales larger than *τ*_*f*_ but much smaller than *τ*_*s*_. On such timescales, transients in the macroscopic concentrations of fast species have decayed, a quasi-steady-state is achieved and by the deterministic QSSA, we know that the fast-species concentrations can be expressed in terms of those of the slow-species concentrations. Now the latter concentrations vary very slowly over timescales much smaller than *τ*_*s*_implying that for all intents and purposes they can be considered constant. Hence the matrices in equation (39) can be considered time-independent. It then follows that the solution to the latter equation is approximately given by

(41) $$\begin{array}{lll} {\vec{\eta }}_{f}(t)\approx & \;e^{(t-t_{0})\tilde{J}\;{\;}_{f}/\tau_{f}}{\vec{\eta }}_{f}(t_{0})\; & \;\\ & +\overset{t}{\underset{t_{0}}{\int }}dt^{\prime }e^{(t-t^{\prime })\tilde{J}\;{\;}_{f}/\tau_{f}}J\;{\;}_{\mathrm{fs}}{\vec{\eta }}_{s}(t^{\prime })\; & \;\\ & +\overset{t}{\underset{t_{0}}{\int }}dt^{\prime }e^{(t-t^{\prime })\tilde{J}\;{\;}_{f}/\tau_{f}}\frac{1}{\sqrt{\Omega }}\;S\;{\;}_{f}\sqrt{F}\;\;\vec{\Gamma }(t^{\prime }),\; \end{array}$$

where we have put $\tilde{J}\;{\;}_{f}=\tau_{f}J\;{\;}_{f}$. In the case when the correlation time is very short the first term can be neglected and the lower limit of the integration in the other terms can be extended to *t*_0_→−*∞*. To make further analytical progress, we switch to Fourier space. First we derive the following result which will prove very useful. Given a vector $\vec{f}(t)$, we have

(42) $$\begin{array}{cc} \;\overset{t}{\underset{-\infty }{\int }}dt^{\prime }e^{(t-t^{\prime })\;J\;{\;}_{f}}\vec{f}(t^{\prime }) & =-\;\overset{\infty }{\underset{-\infty }{\int }}dt^{\prime }\\ \;\;\int \frac{\mathrm{d\omega}}{2\Pi }e^{\mathrm{i\omega}(t-t^{\prime })}\frac{1}{J\;{\;}_{f}-i\;I\;\omega }\\ \;\;\int \frac{d\omega^{\prime }}{2\Pi }e^{i\omega^{\prime }t^{\prime }}\hat{\vec{f}}(\omega^{\prime })\\ =-\int \frac{\mathrm{d\omega}}{2\Pi }e^{\mathrm{i\omega t}} \end{array}$$

(43) $$\int d\omega^{\prime }\delta (\omega -\omega^{\prime })\frac{1}{J\;{\;}_{f}-i\;I\;\omega }\hat{\vec{f}}(\omega^{\prime })$$

(44) $$=-\int \frac{\mathrm{d\omega}}{2\Pi }e^{\mathrm{i\omega t}}\frac{1}{J\;{\;}_{f}-i\;I\;\omega }\hat{\vec{f}}(\omega ),$$

where $\hat{\vec{f}}(\omega )$ denotes the Fourier transform of $\vec{f}(t)$ and I is the identity matrix. It then follows that the Fourier transform of equation (41) is given by

(45) $$\begin{array}{lll} \;{\hat{\vec{\eta }}}_{f}(\omega ) & \approx -\tau_{f}\left(\;{\left(\tilde{J}\;{\;}_{f}-i\;I\;\omega \tau_{f}\right)}^{-1}J\;{\;}_{\mathrm{fs}}{\hat{\vec{\eta }}}_{s}(\omega )\right)\; & \;\\ & \;\left(+{\left(\tilde{J}\;{\;}_{f}\;-\;i\;I\;\omega \tau_{f}\right)}^{-1}\frac{1}{\sqrt{\Omega }}S\;{\;}_{f}\;\sqrt{F}\;\;\hat{\vec{\Gamma }}(\omega )\right).\; \end{array}$$

Since we are interested in a description on timescales larger than *τ*_*f*_, i.e., for fluctuations of frequency $\omega \ll \tau_{f}^{-1}$, then the above equation further reduces to

(46) $${\hat{\vec{\eta }}}_{f}(\omega )\approx -J\;{\;}_{f}^{-1}J{\;}_{\mathrm{fs}}{\hat{\vec{\eta }}}_{s}(\omega )-\frac{1}{\sqrt{\Omega }}J\;{\;}_{f}^{-1}S\;{\;}_{f}\sqrt{F}\;\;\hat{\vec{\Gamma }}(\omega ).$$

Taking the inverse Fourier transform of the above equation and substituting in equation (40) we obtain

(47) $$\begin{array}{lll} \;\frac{d}{\mathrm{dt}}{\vec{\eta }}_{s}= & \;\left(J{\;}_{s}-J{\;}_{\mathrm{sf}}J\;{\;}_{f}^{-1}J\;{\;}_{\mathrm{fs}}\right){\vec{\eta }}_{s}\; & \;\\ & +\frac{1}{\sqrt{\Omega }}\left(S{\;}_{s}-J{\;}_{\mathrm{sf}}J\;{\;}_{f}^{-1}S\;{\;}_{f}\right)\sqrt{F}\;\;\vec{\Gamma }(t).\; \end{array}$$

This Langevin equation is the ssLNA: it is an effective stochastic description of the intrinsic noise in the slow variables in timescale separation conditions. Using standard methods [37] it can be shown that the FPE which is equivalent to this effective Langevin equation is equation (9). The ssLNA can also be derived more rigorously using the projection operator formalism as shown in [57].

#### A note on the reduced Jacobian of the ssLNA

Here we show that the reduced Jacobian $J=J{\;}_{s}-J{\;}_{\mathrm{sf}}J\;{\;}_{f}^{-1}J\;{\;}_{\mathrm{fs}}$ in the ssLNA equation (47) is exactly the Jacobian of the reduced REs which arise from applying the deterministic QSSA on the REs of the full network. One starts by considering a small deviation from the deterministic steady state $[\vec{X}]\to [\vec{X}]+\vec{\Delta }$ on the REs of the full network. Using the partitioned Jacobian of the form as in equation (11), we can then write

(48) $$\begin{array}{lll} & \frac{\partial }{\mathrm{\partial t}}{\vec{\Delta }}_{f}=J\;{\;}_{f}{\vec{\Delta }}_{f}+J\;{\;}_{\mathrm{fs}}{\vec{\Delta }}_{s},\; & \;\\ & \frac{\partial }{\mathrm{\partial t}}{\vec{\Delta }}_{s}=J{\;}_{\mathrm{sf}}{\vec{\Delta }}_{f}+J{\;}_{s}{\vec{\Delta }}_{s},\; \end{array}$$

with slow and fast perturbations ${\vec{\Delta }}_{s}$ and ${\vec{\Delta }}_{f}$, respectively. Applying the deterministic QSSA, i.e., setting the time derivative of fast perturbations to zero, one finds

(49) $$\begin{array}{ll} & \frac{\partial }{\mathrm{\partial t}}{\vec{\Delta }}_{s}=\left(J{\;}_{s}-J{\;}_{\mathrm{sf}}J\;{\;}_{f}^{-1}J\;{\;}_{\mathrm{fs}}\right){\vec{\Delta }}_{s}=J\;\;{\vec{\Delta }}_{s}.\; \end{array}$$

Hence the Jacobian in the ssLNA equations (9) and (12) is the same as the Jacobian of the reduced REs.

Note that equations (48) are formally the same as obtained by taking the average of the LNA equations (39) and (40) (this general agreement between the LNA and linear stability analysis is discussed in [21]). This implies that the timescales of the fast and slow variables in the ssLNA (and hence of the CME under timescale separation conditions and in the macroscopic limit) is the same as the timescales obtained from the REs.

### Details of the derivations for the two-subunit enzyme network

#### The ssLNA recipe: Langevin equation and noise statistics

We here show the details of the ssLNA method as applied to the network discussed in Application I in the Results section. The first step of the recipe is to cast the reaction scheme of the full network (14) into the form of the general reaction scheme (1). This is done by setting *X*_1_=*S*, *X*_2_=*EE*, *X*_3_=*EES* and *X*_4_=*SEES* and by labeling the input reaction as reaction 1, the binding of *S* to *EE* as reaction 2, the decay of *EES* to *S* and *EE* as reaction 3, the decay of *EES* to *EE* and *P* as reaction 4, the binding of *S* to *EES* as reaction 5, the decay of *SEES* into *EES* and *S* as reaction 6 and finally the decay of *SEES* into *EES* and *P* as reaction 7. Note that the reaction number labeling is arbitrary but the labeling of the species is not: according to the convention set out in the Introduction, we have to choose the substrate as the first species because it is the slow variable, while the rest of the species are the fast ones. Given the chosen order of the species and the reactions, the stoichiometric matrix and the macroscopic rate function vector (see definitions in the Background section and the description of the ssLNA in the Results section) are given by (50)

(51) $$\begin{array}{lll} \vec{f} & =\left(k_{\mathrm{in}},k_{1}[S][\mathrm{EE}],k_{-1}[\mathrm{EES}],k_{3}[\mathrm{EES}],\right)\; & \;\\ & \;\left(k_{2}[S][\mathrm{EES}],k_{-2}[\mathrm{SEES}],k_{4}[\mathrm{SEES}]\right).\; \end{array}$$

Note that the row number of the stoichiometric matrix reflects the species number, while the column number reflects the reaction number. The order of the entries in the macroscopic rate function vector reflects the reaction number.

The enzyme can only be in one of three forms, *EE*, *EES* and *SEES* and hence we have the conservation law, $\left[EE_{T}\right]=[\mathrm{EE}]+[\mathrm{EES}]+[\mathrm{SEES}]$, where [*EE*_*T*_] is the total enzyme concentration, which is a time-independent constant. Hence, we are free to remove information from the stoichiometric matrix about one of the enzyme forms; we choose to remove information about *EE*, and therefore, we eliminate the second row from the stoichiometric matrix, leading to (52)

Note that we have also partitioned the stoichiometric matrix into two sub-matrices as required by our method (see prescription for ssLNA in the Results section). Now we can use this matrix together with the macroscopic rate function vector $\vec{f}$ to obtain the elements of the Jacobian matrix ${\left(J\;{\;}_{F}\right)}_{\mathrm{ij}}=\partial_{j}{\left(S\;\;\vec{f}\right)}_{i}$ of the REs of the full network(53)

where we also partitioned the matrix into 4 sub-matrices as required by our formulation of the ssLNA in the Results section. Now we can use the two sub-matrices of the stoichiometric matrix and the four sub-matrices of the Jacobian to calculate the matrix A - B (as given by the two equations for A and B after equation (10)), which yields

(54) $$\begin{array}{lll} \;A-B & =\left(\sqrt{k_{\mathrm{in}}},-q_{1}\sqrt{k_{1}[S][\mathrm{EE}]},q_{1}\sqrt{k_{-1}[\mathrm{EES}]},\right)\; & \;\\ & \;-(1-q_{1})\sqrt{k_{3}[\mathrm{EES}]},-q_{2}\sqrt{k_{2}[S][\mathrm{EES}]},\; & \;\\ & \;\left(q_{2}\sqrt{k_{-2}[\mathrm{SEES}]},-\left(1-q_{2}\right)\sqrt{k_{4}[\mathrm{SEES}]}\right),\; \end{array}$$

where *q*_1_and *q*_2_ are as defined in the main text by equations (19) and (20). Furthermore, the Jacobian of the reduced RE, equation (15) in the main text, is given by

(55) $$J=-\frac{d}{d[S]}\frac{[S]\frac{\left[EE_{T}\right]}{K_{m1}}\left(k_{3}+k_{4}\frac{\left[S\right]}{K_{m2}}\right)}{1+\frac{\left[S\right]}{K_{m1}}+\frac{{\left[S\right]}^{2}}{K_{m1}K_{m2}}}.$$

Finally, the Langevin equation, equation (18), is obtained by substituting equations (55) and (54) in equation (12). The equation for the variance of the substrate fluctuations, equation (21), is obtained by substituting equation (54) in equation (10) to obtain the new diffusion scalar *D*_*ss*_ and then substituting the latter together with the new Jacobian equation (55) in the Lyapunov equation, equation (6), with *D*_*h*_ replaced by *D*_*ss*_. Note that in this example because we have only one slow species, the Lyapunov equation is not a matrix equation but simply a single linear algebraic equation for the variance. For the same reason we have a diffusion scalar rather than a diffusion matrix. The power spectrum can be obtained by substituting the new Jacobian and diffusion scalar in equation (7) (with *D*_*h*_ replaced by *D*_*ss*_), leading to

(56) $$P(\omega )=\frac{\Omega^{-1}\left(\;A-B\;\right){\left(\;A-B\;\right)}^{T}}{\omega^{2}+J^{2}}.$$

The power decays monotonically with frequency, which implies no noise-induced oscillation; this statement is generally true for all networks (full or coarse-grained) which have just one slow species.

#### The hLNA recipe: Langevin equation and noise statistics

Here we apply the LNA to the heuristic CME according to the method described in the Results section. The coarse-grained network is given by reaction scheme (17); an elementary reaction for the substrate input process and a non-elementary first-order reaction for substrate catalysis. The stoichiometric matrix and macroscopic rate function vector are given by

(57) S=(1,−1),

(58) $$\vec{f}=\left(k_{\mathrm{in}},k^{\prime }[S]\right),$$

where *k*^*′*^ is defined in the main text, equation (16). The diffusion scalar *D*_*h*_of the linear FPE approximating the heuristic master equation for this process can be constructed from the stoichiometric and macroscopic rate function matrices using equation (5), which leads to $D_{h}=\Omega^{-1}\left(k_{\mathrm{in}}+k^{\prime }[S]\right)=\Omega^{-1}\left(k_{\mathrm{in}}+k_{3}[\mathrm{EES}]+k_{4}[\mathrm{SEES}]\right)$. Note that here we have a diffusion scalar rather than a matrix because we have only one slow variable. Finally, from equations (6) and (7), the variance and the power spectrum are obtained from the diffusion scalar and the Jacobian, *J*, of the effective rate equation, equation (15), leading to

(59) $$\sigma_{\mathrm{hLNA}}^{2}=H=-\frac{D_{h}}{2J},$$

(60) $$P(\omega )=\frac{D_{h}}{\omega^{2}+J^{2}}.$$

In the Results section, it was shown that a reduced CME description becomes possible whenever the effective stoichiometric matrix S’ as given by equation (13) evaluates to integer values. For the reaction scheme under consideration, it can be shown that $S^{\prime }=\left(1,-q_{1},q_{1},-\left(1-q_{1}\right),-q_{2},q_{2},-\left(1-q_{2}\right)\right)$, where *q*_1_and *q*_2_ are given by equations (19) and (20). The latter two quantities are generally real values and time-dependent and hence a reduced CME description is not generally possible. A simple choice which makes S’ integer-valued is the null choice, *q*_1_=*q*_2_=0, and indeed it is for these values that in the main text we show that the hLNA (and hence the heuristic CME) is a valid description of stochastic kinetics under timescale separation conditions.

### Details of the derivations for the gene network example

#### Reduced rate equations

The fast species of the genetic network with negative feedback given in the main text are given by the gene species *G*, *GP*, *GP*_2_ and the enzyme species *E* and *EP*. There are two conservation laws, $\left[G_{T}\right]=[G]+[\mathrm{GP}]+[GP_{2}]$ for the gene species and $\left[E_{T}\right]=[E]+[\mathrm{EP}]$ for the enzyme species and hence we need to apply the deterministic QSSA only to two of the gene species and to one of the enzyme species. The QSSA applied to the latter is the standard Briggs-Haldane approximation, which is well known [38], and hence here we restrict our presentation to the QSSA on the negative feedback loop. The macroscopic rate equations for the gene species *GP* and *GP*_2_ read

(61) $$\begin{array}{lll} & \;\frac{d[\mathrm{GP}]}{\mathrm{dt}}=k_{1}[G]\;[P]-k_{-1}[\mathrm{GP}]-k_{2}[\mathrm{GP}]\;[P]+k_{-2}[GP_{2}]\;,\; & \;\\ & \;\frac{d[GP_{2}]}{\mathrm{dt}}=k_{2}[\mathrm{GP}][P]-k_{-2}[GP_{2}].\; \end{array}$$

Substituting the gene conservation law, setting the time derivatives to zero and solving these two equations simultaneously, we obtain the quasi-steady-state concentrations of the three gene species

(62) $$\begin{array}{lll} \frac{\left[G\right]}{\left[G_{T}\right]} & =\frac{K^{2}}{K^{2}+K_{2}[P]+{\left[P\right]}^{2}},\; & \;\\ \frac{\left[\mathrm{GP}\right]}{\left[G_{T}\right]} & =\frac{K_{2}[P]}{K^{2}+K_{2}[P]+{\left[P\right]}^{2}},\; & \;\\ \frac{\left[GP_{2}\right]}{\left[G_{T}\right]} & =\frac{{\left[P\right]}^{2}}{K^{2}+K_{2}[P]+{\left[P\right]}^{2}},\; \end{array}$$

where *K*_1_=*k*_−1_/*k*_1_, *K*_2_=*k*_−2_/*k*_2_ and *K*^2^=*K*_1_*K*_2_. Since only the ternary complex (one with 3 molecules, i.e., *GP*_2_) does not lead to mRNA production, the active gene fraction is given by

(63) $$\begin{array}{ll} \frac{\left[G]+[\mathrm{GP}\right]}{\left[G_{T}\right]}=\frac{K^{2}+K_{2}[P]}{K^{2}+K_{2}[P]+{\left[P\right]}^{2}}\to \frac{K^{2}}{K^{2}+{\left[P\right]}^{2}}, & \; \end{array}$$

where in the last step we have drawn the limit of cooperative binding *K*_2_→0 at constant *K* (or equivalently *k*_2_→*∞*, *k*_1_→0 at constant *k*_1_*k*_2_). It follows that the REs for the slow variables of mRNA and protein concentrations are then given by

(64) $$\begin{array}{lll} \frac{d[M]}{\mathrm{dt}} & =\frac{k_{0}\left[G_{T}\right]K^{2}}{K^{2}+{\left[P\right]}^{2}}-k_{\mathrm{dM}}[M],\; & \;\\ \frac{d[P]}{\mathrm{dt}} & =k_{s}[M]-\frac{k_{4}\left[E_{T}\right][P]}{K_{M}+[P]},\; \end{array}$$

where $K_{M}=\left(k_{-3}+k_{4}\right)/k_{3}$ is the Michaelis-Menten constant of the enzyme which degrades the protein species.

#### Derivation of the ssLNA results

We cast the species in the full network (as given by reaction schemes (31) and (32)) into the form required by the convention set in the Introduction. We denote the slow species by *X*_1_=*M* and *X*_2_=*P* and the fast species by *X*_3_=*GP*, *X*_4_=*GP*_2_ and *X*_5_=*EP*. Note that the form of the gene with no bound protein (*G*) as well as the free enzyme species (*E*) do not appear explicitly in our description due to the two inherent conservation laws (same as shown in the previous section for the enzyme example except that here we immediately remove the extra species). The eleven constituent reactions are numbered in the following order: *G*→*G* + *M*, *GP*→*GP* + *M*, *P* + *G*→*GP*, *GP*→*P* + *G*, *P* + *GP*→*GP*_2_, *GP*_2_→*P* + *GP*, *M*→∅, *M*→*M* + *P*, *P* + *E*→*EP*, *EP*→*P* + *E*, and *EP*→*E*.

The stoichiometric matrix and the macroscopic rate function vector are constructed as (65)

(66) $$\begin{array}{lll} \;\vec{f}= & \;\left(k_{0}[G],k_{0}[\mathrm{GP}],k_{1}[P][G],k_{-1}[\mathrm{GP}],k_{2}[P][\mathrm{GP}],\right)\; & \;\\ & k_{-2}[GP_{2}],k_{\mathrm{dM}}[M],k_{s}[M],k_{3}[E][P],k_{-3}[\mathrm{EP}],\; & \;\\ & \left(k_{4}[\mathrm{EP}]\right).\; \end{array}$$

Note that the columns of S reflect the reaction number, while the rows reflect the species number. Similarly, the reaction number is reflected in the entries of the macroscopic rate function vector $\vec{f}$.

From S and $\vec{f}$ we can obtain the Jacobian matrix, ${\left(J\;{\;}_{F}\right)}_{\mathrm{ij}}=\partial_{j}{\left(S\;\;\vec{f}\right)}_{i}$, of the REs of the full network (67)

where the individual submatrices read explicitly

(68) $${\underset{─}{J}}_{f}\;=\;\left[\;\begin{array}{ccc} \;-k_{-1}\;-\;(k_{1}\;+\;k_{2})[P] & \;k_{-2}\;-k_{1}[\;P] & 0\\ k_{2}[\;P] & \;-k_{-2} & 0\\ 0 & 0 & \;-k_{4}\;-\;k_{-3}\;-\;k_{3}[\;P] \end{array}\;\right]\;,$$

(69) $$J_{s}=\left[\begin{array}{cc} -k_{dM} & 0\\ k_{s} & -[G]k_{1}-[GP]k_{2}-[E]k_{3} \end{array}\right],$$

(70) $$J_{sf}=\left[\begin{array}{ccc} 0 & -k_{0} & 0\\ k_{-1}+k_{1}[P]-k_{2}[P] & k_{-2}+k_{1}[P] & k_{-3}+k_{3}[P] \end{array}\right],$$

(71) $$J\;{\;}_{fs}=\left[\begin{array}{ll} 0 & k_{1}[G]-k_{2}[GP]\\ 0 & \;k_{2}[GP]\\ 0 & \;k_{3}[E] \end{array}\right].$$

Using these Jacobian submatrices, the stoichiometric submatrices given in equation (65) and the diagonal matrix F whose elements are those of the macroscopic rate function vector $\vec{f}$, as given in equation (66), we obtain (using the two equations for A and B after equation (10)) the matrix

(72) $$\begin{array}{cc} \;{\left(\;A-B\;\right)}^{T}=\left[\begin{array}{cc} \sqrt{k_{0}[G]} & 0\\ \sqrt{k_{0}[GP]} & 0\\ -p_{1}\sqrt{k_{1}[G][P]} & 0\\ p_{1}\sqrt{k_{-1}[GP]} & 0\\ -p_{2}\sqrt{k_{2}[GP][P]} & 0\\ +p_{2}\sqrt{k_{-2}[GP_{2}]} & 0\\ -\sqrt{k_{dM}[M]} & 0\\ 0 & \sqrt{k_{s}[M]}\\ 0 & -(1-q)\sqrt{k_{3}[E][P]}\\ 0 & (1-q)\sqrt{k_{-3}[EP]}\\ 0 & -q\sqrt{k_{4}[EP]} \end{array}\right], & \; \end{array}$$

where

(73) $$p_{1}=\frac{k_{0}}{k_{1}}\frac{\left[P\right]}{K^{2}+K_{2}[P]+{\left[P\right]}^{2}}\overset{K_{2}\to 0}{\to }\frac{k_{0}}{k_{1}}\frac{\left[P\right]}{K^{2}+{\left[P\right]}^{2}},$$

(74) $$p_{2}=\frac{k_{0}}{k_{2}}\frac{K_{1}+[P]}{K^{2}+K_{2}[P]+{\left[P\right]}^{2}}\overset{k_{2}\to \infty }{\to }0,$$

(75) $$q=\frac{[P]+K_{3}}{[P]+K_{M}}.$$

Note that *K*_3_=*k*_−3_/*k*_3_. The Jacobian can be obtained from the reduced REs, equation (64), and is given by

(76) $$\begin{array}{cc} J & =\left[\begin{array}{cc} -k_{\mathrm{dM}} & -\frac{2k_{0}\left[G_{T}\right]K^{2}[P]}{{\left(K^{2}+{\left[P\right]}^{2}\right)}^{2}}\\ k_{s} & -\frac{\left[E_{T}\right]k_{4}K_{M}}{{\left(K_{M}+[P]\right)}^{2}} \end{array}\right].\; \end{array}$$

Note that we have drawn the limit of cooperative binding on *p*_1_, *p*_2_, *q* and J.

Finally the Langevin equation is obtained by substituting equations (76) and (72) in equation (12). To obtain the equation for the variance of the mRNA and protein fluctuations, one must first determine the diffusion matrix D_*ss*_. Using equation (72) and the definition (10), it can be readily shown that the diffusion matrix takes the diagonal form

(77) $$\begin{array}{lll} D{\;}_{\mathrm{ss}} & =\Omega^{-1}\text{diag}\left(D_{M},D_{P}\right),\; & \;\\ D_{M} & =k_{\mathrm{dM}}[M]+\frac{k_{0}\left[G_{T}\right]K^{2}}{K^{2}+{\left[P\right]}^{2}}+p_{1}\frac{2k_{0}\left[G_{T}\right]K^{2}{\left[P\right]}^{2}}{{\left(K^{2}+{\left[P\right]}^{2}\right)}^{2}},\; & \;\\ D_{P} & =k_{s}[M]+\frac{k_{4}\left[E_{T}\right][P]}{K_{M}+[P]}-(1-q)\frac{2k_{4}\left[E_{T}\right]{\left[P\right]}^{2}}{{\left(K_{M}+[P]\right)}^{2}}.\; \end{array}$$

The covariance matrix equation can then be obtained by substituting the new diffusion matrix, equation (77), together with the Jacobian matrix, equation (76), in the Lyapunov equation (6) with D_*h*_ replaced by D_*ss*_. Note that unlike the enzyme kinetics example, in the gene network example, we have two slow species, and hence the Lyapunov equation is a matrix equation involving the simultaneous solution of two linear equations. The explicit equations for the variances of the mRNA and protein fluctuations about the macroscopic steady-state concentrations are the diagonal elements of the covariance matrix H, which are found to be

(78) $$\begin{array}{lll} \sigma_{M}^{2} & =H_{11}=\frac{-\left(\mathrm{Det}\;J+J_{22}^{2}\right)D_{M}-J_{12}^{2}D_{P}}{2\;\mathrm{Tr}\;J\;\mathrm{Det}\;J\;\Omega },\; & \;\\ \sigma_{P}^{2} & =H_{22}=\frac{J_{21}^{2}D_{M}-\left(\mathrm{Det}\;J+J_{11}^{2}\right)D_{P}}{2\;\mathrm{Tr}\;J\;\mathrm{Det}\;J\;\Omega },\; \end{array}$$

where Det J and Tr J refer to the determinant and trace of the Jacobian matrix J, respectively. Similarly, the power spectra of the fluctuations are obtained by substituting the new diffusion matrix, equation (77), together with the Jacobian matrix, equation (76), in equation (7) (with D_*h*_ replaced by D_*ss*_), leading to

(79) $$\begin{array}{lll} P_{M}(\omega ) & =\frac{\left(J_{22}^{2}+\omega^{2}\right)D_{M}+J_{12}^{2}D_{P}}{\Omega \left[{\left(\mathrm{Det}\;J\;\right)}^{2}+\left({\left(\mathrm{Tr}\;J\;\right)}^{2}-2\mathrm{Det}\;J\;\right)\omega^{2}+\omega^{4}\right]},\; & \;\\ P_{P}(\omega ) & =\frac{J_{21}^{2}D_{M}+\left(J_{11}^{2}+\omega^{2}\right)D_{P}}{\Omega \left[{\left(\mathrm{Det}\;J\;\right)}^{2}+\left({\left(\mathrm{Tr}\;J\;\right)}^{2}-2\mathrm{Det}\;J\;\right)\omega^{2}+\omega^{4}\right]}.\; \end{array}$$

It can be shown that the condition to observe a peak in the mRNA power spectrum is given by [35]:

(80) $$\begin{array}{ll} \;\left(J_{22}^{2}D_{M}+J_{12}^{2}D_{P}\right)\left({\left(\mathrm{Tr}\;J\;\right)}^{2}-2\mathrm{Det}\;J\;\right)-D_{M}\;{\left(\mathrm{Det}\;J\;\right)}^{2}<0. & \; \end{array}$$

#### Derivation of the hLNA results

An inspection of the reduced REs, equations (64), shows that the coarse-grained network is composed of 4 reactions, two elementary and two non-elementary with a stoichiometry matrix and a macroscopic rate function vector given by

(81) $$\begin{array}{l} S=\left[\begin{array}{llll} +1 & -1 & 0 & 0\\ 0 & 0 & +1 & -1 \end{array}\right],\\ \vec{f}=\left(\frac{k_{0}\left[G_{T}\right]K^{2}}{K^{2}+{\left[P\right]}^{2}},k_{\mathrm{dM}}[M],k_{s}[M],\frac{k_{4}\left[E_{T}\right][P]}{K_{M}+[P]}\right), \end{array}$$

where we denoted the mRNA as species 1 and the protein as species 2. These can be used to calculate the diffusion matrix of the hLNA using equation (5), which leads to

(82) $$\begin{array}{lll} D{\;}_{h} & =\Omega^{-1}\text{diag}\left(D_{h,M},D_{h,P}\right),\; & \;\\ D_{h,M} & =k_{\mathrm{dM}}[M]+\frac{k_{0}\left[G_{T}\right]K^{2}}{K^{2}+{\left[P\right]}^{2}},\; & \;\\ D_{h,P} & =k_{s}[M]+\frac{k_{4}\left[E_{T}\right][P]}{K_{M}+[P]}.\; \end{array}$$

The covariance matrix and the spectra can be obtained as for the ssLNA. The variances and spectra are given by equation (78) and equation (79) with *D*_*M*_replaced by *D*_*h*,*M*_, and *D*_*P*_ replaced by *D*_*h*,*P*_.

In the main text, we show that the hLNA (and hence the heuristic CME) is the correct stochastic description under timescale separation when *p*_1_=0and *q*=1. Indeed one finds that this choice satisfies the condition derived in the Methods section, which is necessary to have a reduced CME description under timescale separation conditions. Namely the choice *p*_1_=0 and *q*=1 forces the effective stoichiometric matrix S’ given by equation (13) to assume strictly integer values.

### Comparison with other stochastic model reduction methods

In this section, we compare the predictions of the ssLNA with the predictions of other stochastic model reduction techniques in the literature. Specifically, we compare with the recent methods of Pahlajani *et al.*[51] and of Shahrezaei and Swain [55].

We consider a simple model of stochastic gene expression given by

(83) $$\begin{array}{lll} G\overset{k_{0}}{\to }G+M,\; & M\overset{k_{\mathrm{dM}}}{\to }\emptyset ,\; & \;\\ M\overset{k_{s}}{\to }M+P,\; & P\overset{k_{\mathrm{dP}}}{\to }\emptyset ,\; \end{array}$$

which describes transcription, translation and degradation of mRNA and protein. The deterministic REs for this example read

(84) $$\frac{d}{\mathrm{dt}}[M]=k_{0}\left[G_{T}\right]-k_{\mathrm{dM}}[M],$$

(85) $$\frac{d}{\mathrm{dt}}[P]=k_{s}[M]-k_{\mathrm{dP}}[P].$$

In the common case where the mRNA timescale is very small compared to that of protein, i.e., $\gamma =\left(k_{\mathrm{dM}}/k_{\mathrm{dP}}\right)\gg 1$, the mRNA concentration will quickly relax to its steady state value $[M]=k_{0}\left[G_{T}\right]/k_{\mathrm{dM}}$ and hence the REs can be reduced to

(86) $$\begin{array}{ll} & \frac{d}{\mathrm{dt}}[P]=k_{0}\left[G_{T}\right]b-k_{\mathrm{dP}}[P],\; \end{array}$$

where the parameter *b*=*k*_*s*_/*k*_dM_has been interpreted as the burst size (the average number of proteins synthesized per mRNA transcript) [56]. This is the deterministic QSSA.

Shahrezaei and Swain [55] showed that in the same limit of time scale separation, one can obtain the exact joint probability distribution of mRNA and protein fluctuations by solving the generating function equation associated with the CME of the full network. The variance of protein concentration fluctuations in steady-state conditions can be calculated from this distribution function and was found to be given by

(87) $$\begin{array}{ll} \langle \eta_{P}^{2}\rangle =\frac{1}{\Omega }[P]\left(1+b\right). & \; \end{array}$$

The ssLNA gives the following Langevin equation description of the system

(88) $$\begin{array}{lll} \;\frac{d}{\mathrm{dt}}\eta_{P}= & -k_{\mathrm{dP}}\eta_{P}+b\;\sqrt{\frac{\left[G_{T}\right]k_{0}}{\Omega }}\Gamma_{1}(t)-b\;\sqrt{\frac{k_{\mathrm{dM}}[M]}{\Omega }}\Gamma_{2}(t)\; & \;\\ & +\sqrt{\frac{k_{s}[M]}{\Omega }}\Gamma_{3}(t)-\sqrt{\frac{k_{\mathrm{dP}}[P]}{\Omega }}\Gamma_{4}(t).\; \end{array}$$

The steady state variance predicted by the above Langevin equation is given by equation (87). The same result has also been previously obtained by Paulsson [32] by applying the LNA to the full network given by (83) and subsequently taking the limit of timescale separation. Hence, the result obtained from the ssLNA agrees with the exact method of Shahrezaei and Swain. The advantage of the ssLNA is that it is generally applicable to arbitrarily complex biochemical networks, whereas the generating function method of solving CMEs is typically restricted to networks composed of at most first-order reactions or very simple bimolecular reactions [18,19].

Recently, another approximate reduction technique based on the LNA has been proposed by Pahlajani, Atzberger and Khammash [51]. The authors utilize the assumption that in the limit of timescale separation, the diffusion matrix of the full network can be decomposed in block diagonal form as

(89) $$\begin{array}{l} D=\left[\begin{array}{ll} D{\;}_{s} & 0\\ 0 & D_{f} \end{array}\right], \end{array}$$

where D_*f *_ is of order *γ*^−1^and *γ* is a large parameter under timescale separation conditions. This leads to a FPE for the slow variables with reduced Jacobian and diffusion matrices given by

(90) $$J=\;J{\;}_{s}-J{\;}_{\mathrm{sf}}\;J\;{\;}_{f}^{-1}\;J\;{\;}_{\mathrm{fs}},$$

(91) $$D=\;D{\;}_{s}=\Omega^{-1}\;S{\;}_{s}\;F\;\;S{\;}_{s}^{T}.$$

The authors showed that the application of this formalism to the gene example above, leads to a Langevin equation of the form

(92) $$\begin{array}{ll} \frac{d}{\mathrm{dt}}\eta_{P}=-k_{\mathrm{dP}}\eta_{P}+\sqrt{\frac{k_{s}[M]}{\Omega }}\Gamma_{3}(t)-\sqrt{\frac{k_{\mathrm{dP}}[P]}{\Omega }}\Gamma_{4}(t). & \; \end{array}$$

The variance of fluctuations predicted by the above Langevin equation is given by equation (87) with *b*≪1. Hence, it is clear that the method of Pahlajani *et al.* cannot capture the fluctuations about the steady-state concentrations for all choices of rate constants which are compatible with the deterministic QSSA. Rather their assumption regarding the form of the diffusion matrix limits their analysis to a subset of the parameter space over which the deterministic QSSA and consequently the ssLNA are valid. Indeed, the fact that the method by Pahlajani *et al.* is generally a special case of the ssLNA can also be seen by direct comparison of the diffusion matrices of the two methods, namely, equations (91) and (10).

## Competing interests

The authors declare that they have no competing interests.

## Authors’ contributions

PT developed the mathematical formulation of the ssLNA and performed the stochastic simulations to corroborate its predictions. AVS contributed to the interpretation of the derivations, in particular to the clarification of issues concerning timescale separation. RG supervised the research, contributed to the derivation of the implicit assumptions of the hLNA and to derivations concerned with the Langevin formulation of the ssLNA, and wrote the manuscript. All authors read and approved the final manuscript.

## References

[B1] Schwikowski B Uetz P Fields S A network of protein-protein interactions in yeast Nat Biotechnol 2000 18 12 1257 1261 11101803 10.1038/82360

[B2] Ghaemmaghami S Huh W Bower K Howson R Belle A Dephoure N O’Shea E Weissman J Global analysis of protein expression in yeast Nature 2003 425 6959 737 741 14562106 10.1038/nature02046

[B3] Ishihama Y Schmidt T Rappsilber J Mann M Hartl F Kerner M Frishman D Protein abundance profiling of the Escherichia coli cytosol BMC Genomics 2008 9 102 18304323 10.1186/1471-2164-9-102 PMC2292177

[B4] Gillespie D Exact stochastic simulation of coupled chemical reactions J Phys Chem 1977 81 25 2340 2361

[B5] Gillespie D Stochastic simulation of chemical kinetics Annu Rev Phys Chem 2007 58 35 55 17037977 10.1146/annurev.physchem.58.032806.104637

[B6] Segel L Slemrod M The quasi-steady-state assumption: a case study in perturbation SIAM Rev 1989 31 3 446 477

[B7] Gillespie D A rigorous derivation of the chemical master equation Physica A: Stat Mech Appl 1992 188 1-3 404 425

[B8] Janssen J The elimination of fast variables in complex chemical reactions. III. Mesoscopic level (irreducible case) J Stat Phys 1989 57 187 198

[B9] Thomas P Straube A Grima R Limitations of the stochastic quasi-steady-state approximation in open biochemical reaction networks J Chem Phys 2011 135 181103 22088045 10.1063/1.3661156

[B10] Maienschein-Cline M Warmflash A Dinner A Defining cooperativity in gene regulation locally through intrinsic noise Syst Biol, IET 2010 4 6 379 392 10.1049/iet-syb.2009.0070 PMC3390944 21073237

[B11] Assaf M Roberts E Luthey-Schulten Z Determining the stability of genetic switches: explicitly accounting for mRNA noise Phys Rev Lett 2011 106 24 248102 21770603 10.1103/PhysRevLett.106.248102

[B12] Gonze D Hafner M Positive feedbacks contribute to the robustness of the cell cycle with respect to molecular noise Lecture Notes Control Inf Sci 407 283 295 [http://www.springerlink.com/content/w46v57t746564270/]

[B13] Giampieri E Remondini D de Oliveira L Castellani G Lió P Stochastic analysis of a miRNA–protein toggle switch Mol BioSyst 2011 7 10 2796 2803 21717010 10.1039/c1mb05086a

[B14] Rao C Arkin A Stochastic chemical kinetics and the quasi-steady-state assumption: application to the Gillespie algorithm J Chem Phys 2003 118 4999

[B15] Gonze D Halloy J Goldbeter A Deterministic versus stochastic models for circadian rhythms J Biol Phys 2002 28 4 637 653 10.1023/A:1021286607354 PMC3456469 23345804

[B16] Gonze D Abou-Jaoudé W Ouattara D Halloy J How molecular should your molecular model be? On the level of molecular detail required to simulate biological networks in systems and synthetic biology Methods Enzymol 2011 487 171 215 21187226 10.1016/B978-0-12-381270-4.00007-X

[B17] Sanft K Gillespie D Petzold L Legitimacy of the stochastic Michaelis-Menten approximation Syst Biol, IET 2011 5 58 69 10.1049/iet-syb.2009.0057 21261403

[B18] McQuarrie D Stochastic approach to chemical kinetics J Appl Probability 1967 4 3 413 478

[B19] Darvey IG Ninham BW Staff PJ Stochastic models for second order chemical reaction kinetics The Equilibrium State J Chem Phys 1966 45 2145

[B20] Laurenzi I An analytical solution of the stochastic master equation for reversible bimolecular reaction kinetics J Chem Phys 2000 113 3315

[B21] Van Kampen N Stochastic Processes in Physics and Chemistry 2007 Elsevier Science & Technology, Amsterdam

[B22] Grima R Construction and accuracy of partial differential equation approximations to the chemical master equation Phys Rev E 2011 84 056109 10.1103/PhysRevE.84.056109 22181475

[B23] Elf J Ehrenberg M Fast evaluation of fluctuations in biochemical networks with the linear noise approximation Genome Res 2003 13 11 2475 2484 14597656 10.1101/gr.1196503 PMC403767

[B24] Grima R An effective rate equation approach to reaction kinetics in small volumes: Theory and application to biochemical reactions in nonequilibrium steady-state conditions J Chem Phys 2010 133 035101 20649359 10.1063/1.3454685

[B25] Paulsson J Summing up the noise in gene networks Nature 2004 427 6973 415 418 14749823 10.1038/nature02257

[B26] Tao Y Jia Y Dewey T Stochastic fluctuations in gene expression far from equilibrium: Ω expansion and linear noise approximation J Chem Phys 2005 122 124108 15836370 10.1063/1.1870874

[B27] Elf J Ehrenberg M Near-critical behavior of aminoacyl-tRNA pools in E. coli at rate-limiting supply of amino acids Biophys J 2005 88 132 146 15501947 10.1529/biophysj.104.051383 PMC1304992

[B28] Ziv E Nemenman I Wiggins C Optimal signal processing in small stochastic biochemical networks PLoS One 2007 2 10 e1077 17957259 10.1371/journal.pone.0001077 PMC2034356

[B29] Komorowski M Finkenstädt B Harper C Rand D Bayesian inference of biochemical kinetic parameters using the linear noise approximation BMC Bioinf 2009 10 343 10.1186/1471-2105-10-343 PMC2774326 19840370

[B30] Martínez M Soriano J Tlusty T Pilpel Y Furman I Messenger RNA fluctuations and regulatory RNAs shape the dynamics of a negative feedback loop Phys Rev E 2010 81 3 031924 10.1103/PhysRevE.81.031924 20365787

[B31] Keizer J Statistical Thermodynamics of Nonequilibrium Processes 1987 Springer, Berlin

[B32] Paulsson J Models of stochastic gene expression Phys Life Rev 2005 2 2 157 175

[B33] Bar-Even A Paulsson J Maheshri N Carmi M O’Shea E Pilpel Y Barkai N Noise in protein expression scales with natural protein abundance Nat Genet 2006 38 6 636 643 16715097 10.1038/ng1807

[B34] Taniguchi Y Choi PJ Li GW Chen H Babu M Hearn J Emili A Xie X Quantifying E. Coli Proteome and Transcriptome with Single-Molecule Sensitivity in Single Cells Science 2010 329 5991 533 538 20671182 10.1126/science.1188308 PMC2922915

[B35] McKane A Nagy J Newman T Stefanini M Amplified biochemical oscillations in cellular systems J Stat Phys 2007 128 165 191

[B36] Novak B Tyson J Design principles of biochemical oscillators Nat Rev Mol Cell Biol 2008 9 12 981 991 18971947 10.1038/nrm2530 PMC2796343

[B37] Gardiner CW Stochastic Methods: A Handbook for the Natural and Social Sciences 2009 Springer, Berlin

[B38] Fersht A Structure and Mechanism in Protein Science 1999 W.H. Freeman, New York

[B39] Fall C Marland E Wagner J Tyson J Computational Cell Biology 2002 Springer, Berlin

[B40] Selkov E Self-oscillations in glycolysis. 1. A simple kinetic model Eur J Biochem 1968 4 79 86 4230812 10.1111/j.1432-1033.1968.tb00175.x

[B41] Goldbeter A Mechanism for oscillatory synthesis of cyclic AMP in Dictyostelium discoideum Nature 1975 253 540 542 163974 10.1038/253540a0

[B42] Lewis J Autoinhibition with transcriptional delay: A simple mechanism for the zebrafish somitogenesis oscillator Curr Biol 2003 13 16 1398 1408 12932323 10.1016/s0960-9822(03)00534-7

[B43] Tyson J Hong C Dennis Thron C Novak B A simple model of circadian rhythms based on dimerization and proteolysis of PER and TIM Biophys J 1999 77 5 2411 2417 20540926 10.1016/S0006-3495(99)77078-5 PMC1300518

[B44] Rao C Wolf D Arkin A Control, exploitation and tolerance of intracellular noise Nature 2002 420 6912 231 237 12432408 10.1038/nature01258

[B45] Becskei A Serrano L Engineering stability in gene networks by autoregulation Nature 2000 405 6786 590 593 10850721 10.1038/35014651

[B46] McKane A Newman T Predator-prey cycles from resonant amplification of demographic stochasticity Phys Rev Lett 2005 94 21 218102 16090353 10.1103/PhysRevLett.94.218102

[B47] Grima R Noise-induced breakdown of the Michaelis-Menten equation in steady-state conditions Phys Rev Lett 2009 102 21 218103 19519139 10.1103/PhysRevLett.102.218103

[B48] Grima R Investigating the robustness of the classical enzyme kinetic equations in small intracellular compartments BMC Syst Biol 2009 3 101 19814817 10.1186/1752-0509-3-101 PMC2778647

[B49] Thomas P Straube A Grima R Stochastic theory of large-scale enzyme-reaction networks: Finite copy number corrections to rate equation models J Chem Phys 2010 133 195101 21090871 10.1063/1.3505552

[B50] Horsthemke W Brenig L Non-linear Fokker-Planck equation as an asymptotic representation of the master equation Zeitschrift für Physik B: Condensed Matter 1977 27 4 341 348

[B51] Pahlajani CD Atzberger P Khammash M Stochastic reduction method for biological chemical kinetics using time-scale separation J Theor Biol 2011 272 96 112 21126524 10.1016/j.jtbi.2010.11.023

[B52] Zeron ES Santillan M Distributions for negative-feedback-regulated stochastic gene expression: Dimension reduction and numerical solution of the chemical master equation J Theor Biol 2010 264 2 377 385 20144620 10.1016/j.jtbi.2010.02.004

[B53] Cao Y Gillespie DT Petzold L The slow-scale stochastic simulation algorithm J Chem Phys 2005 122 014116 10.1063/1.1824902 15638651

[B54] Mastny E Haseltine E Rawlings J Two classes of quasi-steady-state model reductions for stochastic kinetics J Chem Phys 2007 127 094106 17824731 10.1063/1.2764480

[B55] Shahrezaei V Swain P Analytical distributions for stochastic gene expression Proc Natl Acad Sci 2008 105 45 17256 17261 18988743 10.1073/pnas.0803850105 PMC2582303

[B56] Ozbudak E Thattai M Kurtser I Grossman A van Oudenaarden A Regulation of noise in the expression of a single gene Nat Genet 2002 31 69 73 11967532 10.1038/ng869

[B57] Thomas P Grima R Straube AV Rigorous elimination of fast stochastic variables from the linear noise approximation using projection operators Phys Rev E 2012 86 4 041110 10.1103/PhysRevE.86.041110 23214532

